# A pivotal role for Interferon-α receptor-1 in neuronal injury induced by HIV-1

**DOI:** 10.1186/s12974-020-01894-2

**Published:** 2020-07-29

**Authors:** Hina Singh, Daniel Ojeda-Juárez, Ricky Maung, Rohan Shah, Amanda J. Roberts, Marcus Kaul

**Affiliations:** 1 Division of Biomedical Sciences, School of Medicine, University of California, Riverside, CA 92521 USA; 2 Infectious and Inflammatory Disease Center, Sanford Burnham Prebys Medical Discovery Institute, 10901 North Torrey Pines Road, La Jolla, CA 92037 USA; 3 Animal Models Core, The Scripps Research Institute, 10550 North Torrey Pines Road, MB6, La Jolla, CA 92037 USA

## Abstract

**Background:**

HIV-1 infection remains a major public health concern despite effective combination antiretroviral therapy (cART). The virus enters the central nervous system (CNS) early in infection and continues to cause HIV-associated neurocognitive disorders (HAND). The pathogenic mechanisms of HIV-associated brain injury remain incompletely understood. Since HIV-1 activates the type I interferon system, which signals via interferon-α receptor (IFNAR) 1 and 2, this study investigated the potential role of IFNAR1 in HIV-induced neurotoxicity.

**Methods:**

We cross-bred HIVgp120-transgenic (tg) and IFNAR1 knockout (IFNAR1KO) mice. At 11–14 months of age, we performed a behavioral assessment and subsequently analyzed neuropathological alterations using deconvolution and quantitative immunofluorescence microscopy, quantitative RT-PCR, and bioinformatics. Western blotting of brain lysates and an in vitro neurotoxicity assay were employed for analysis of cellular signaling pathways.

**Results:**

We show that IFNAR1KO results in partial, sex-dependent protection from neuronal injury and behavioral deficits in a transgenic model of HIV-induced brain injury. The IFNAR1KO rescues spatial memory and ameliorates loss of presynaptic terminals preferentially in female HIVgp120tg mice. Similarly, expression of genes involved in neurotransmission reveals sex-dependent effects of IFNAR1KO and HIVgp120. In contrast, IFNAR1-deficiency, independent of sex, limits damage to neuronal dendrites, microgliosis, and activation of p38 MAPK and restores ERK activity in the HIVgp120tg brain. In vitro, inhibition of p38 MAPK abrogates neurotoxicity caused similarly by blockade of ERK kinase and HIVgp120.

**Conclusion:**

Our findings indicate that IFNAR1 plays a pivotal role in both sex-dependent and independent processes of neuronal injury and behavioral impairment triggered by HIV-1.

## Background

HIV-1 infection remains a major public health concern despite effective combination antiretroviral therapy (cART) [1–3]. HIV-1 enters the central nervous system (CNS) early on and continues to cause HIV-associated neurocognitive disorders (HAND) which remain one of the independent risk factors for death due to HIV infection [2, 4, 5]. The pathogenic mechanism of HAND and HIV-associated dementia (HAD), the most severe neurological complication of HIV/AIDS, remains incompletely understood, and there is no specific treatment available [6, 7].

HIV primarily infects CD4^+^ cells of the immune system, such CD4^+^ T cells and monocytes/macrophages and microglia in the CNS [8]. Immune-activated, infiltrating macrophages and resident microglia can harbor HIV in the CNS and start producing neurotoxins, such as excitatory amino acids, arachidonic acid derivatives, free radicals, and pro-inflammatory cytokines [9, 10]. These factors induce neuronal injury, including dendritic and synaptic damage, and eventually apoptosis in the frontal cortex, hippocampus, substantia nigra, putamen, basal ganglia, and cerebellum [8, 11–14]. The key neuropathological features observed in NeuroHIV are astrocytosis, microgliosis, multinucleated giant cells, infiltration of macrophages, decreased synaptic and dendritic density, and frank loss of neuronal cells [8, 11–18].

One model that shares key features of neuropathology, differential gene expression, and impaired learning and memory with people living with HIV (PLWH) and HAND/HAD is a transgenic (tg) mouse expressing the HIV envelope glycoprotein gp120 (HIVgp120) in the CNS [15, 18–20]. HIVgp120tg mice express the soluble viral envelope gp120 of HIV-1 LAV under the control of the glial fibrillary acidic protein (GFAP) promoter in astrocytes [19], and recent studies conducted in our lab revealed that the brains of HIVgp120tg mice show increased expression of interferon-stimulated genes (ISGs) [15, 16]. Interferons (IFNs) are signaling proteins produced and released by host cells in response to pathogens, including viruses, bacteria, parasites, and tumor cells [21]. Since the blood-brain barrier (BBB) restricts access of T and B cells to the brain, the burden of HIV control largely rests with local innate immune defense mechanisms [22]. As a result, IFNs play a major role as the first line of host defense against HIV in the brain [23].

The type I IFNs (IFNα/β) bind to a specific IFN-α/β receptor (IFNAR) that consists of IFNAR1 and IFNAR2 chains [24]. Both IFNα and IFNβ exert their effects by signaling in an autocrine and paracrine manner through the JAK/STAT pathway to activate ISGs [25, 26]. While higher levels of IFNα in the HIV-infected brain correlate with cognitive problems, IFNβ has been reported to control HIV and simian immunodeficiency virus (SIV) infection in the CNS [27–30]. Moreover, we recently demonstrated that IFNβ treatment prevented in vitro and in vivo neuronal injury induced by HIVgp120, depending on the presence of IFNAR1 and CCL 4[16]. However, without the application of exogenous IFNβ, HIVgp120tg mice develop neuropathology, raising the question of whether or not IFNAR1 can contribute to brain injury in the presence of baseline amounts of type I IFNs. Therefore, we investigated in the present study the potential role of type 1 IFN receptor (IFNAR1 subunit) in HIV-induced neurotoxicity.

Here, we report that genetic knock-out of IFNAR1 provides partial protection against HIVgp120-induced neuropathology and behavioral impairment in a sex-dependent fashion. In contrast, the associated cellular signaling via mitogen-activated protein kinases (MAPK) and signal transducer and activator of transcription-1 (STAT1) shows sex-independent effects of HIVgp120 and genetic knockout of IFNAR1 (IFNAR1KO). However, expression of genes involved in neurotransmission and bioinformatics analysis provides further evidence of sexual dimorphism. Overall, our findings suggest that IFNAR1 plays a pivotal role in distinct sex-dependent and independent processes of neuronal injury and behavioral impairment triggered by HIV-1.

## Methods

### Mice

HIVgp120tg mice were kindly provided by Dr. Lennart Mucke (Gladstone Institute of Neurological Disease, University of California, San Francisco, CA) [19], and mice deficient in functional IFNAR1 (B6.129S2-*Ifnar1*^*tm1Agt*^) [31] were provided by Dr. Carl Ware (Sanford Burnham Prebys Medical Discovery Institute, La Jolla, CA). IFNAR1KO and HIV gp120tg mice were crossbred, and the F3 generation of the HIVgp120tg^het^-IFNAR1^het^ mice was used to obtain the following four genotypes: (1) IFNAR1KO-gp120 (IFNAR1^-/-^gp120^+^), (2) HIVgp120tg (IFNAR1^+/+^gp120^+^), (3) IFNAR1KO (IFNAR1^-/-^gp120^-^; control), and (4) WT (IFNAR1^+/+^gp120^-^; wildtype control). All animals were maintained in a mixed C57BL/6.129/SJL genetic background. Genotyping of mice was performed using genomic DNA isolated from tail clippings as previously published in the literature [15, 19] and by The Jackson Laboratory. In the present study, both male and female mice of all four genotypes were analyzed at the age of 11–14 months.

The preparation of cerebrocortical cell cultures was performed as described earlier [17, 32–34]. Briefly, cerebrocortical cells isolated from embryos of E16 Sprague Dawley rats (Harlan Sprague Dawley Inc., San Diego, CA) were plated on Poly-l-lysine-coated glass coverslips in 35-mm plastic dishes and generally used for experiments between 17 and 23 days in vitro when the cultures contained ~30% neurons, ~70% astrocytes, and ~0.1 to 1% microglia [17, 32–34].

All experimental procedures and protocols involving animals were performed in compliance with the National Institute of Health (NIH) guidelines and approved by the Institutional Animal Care and Use Committees (IACUC) of the Sanford Burnham Prebys Medical Discovery Institute (SBP, The Scripps Research Institute (TSRI) and the University of California Riverside (UCR).

### Behavioral assessments

Eleven to 14-month-old WT (male: 11, female: 11), HIVgp120tg (male: 9, female: 8), IFNAR1KO (male: 9, female: 11), and IFNAR1KO × gp120 mice (male: 13, female: 14) were behaviorally tested by the Animal Models Core Facility of TSRI. Mice were housed in standard plastic cages on a reversed 12-h light/dark period (lights on at 8:00 PM), with food and water available ad libitum. The behavioral test battery was designed to examine cognitive abilities as well as general activity, anxiety-like behavior, and visual ability. The order of assessments was light/dark transfer (LDT), locomotor activity (LMA), optomotor (OM), novel object recognition (NOR), and Barnes maze test (BM) and was designed to reduce the potential for confounds due to previous experience. Behavioral testing occurred between 9:00 AM and 12:00 PM (active phase) with 5–7 days between tests. LDT, LMA, OM, and BM were performed as previously published with a minor modification in the BM tests [15, 18]. The NOR test assays recognition memory while leaving the spatial location of the objects intact and is believed to involve the hippocampus, perirhinal cortex, and raphe nuclei [35–37]. Mice were tested with two identical objects placed in the field for 5 min. After three such trials (each separated by 1 min in a holding cage), one of the familiar objects was changed for a novel object. Habituation to the objects across the familiarization trials (decreased contacts) was an initial measure of learning and then renewed interest (increased contacts) in the new object indicated successful object memory.

### Immunofluorescence staining, microscopy, and analysis

The harvest of the brain tissue, immunofluorescence staining, deconvolution and quantitative fluorescence microscopy, and cell counting was performed as recently described [15, 16, 18]. In brief, mice were anesthetized using isoflurane and immediately transcardially perfused with 0.9% saline. The brains were quickly removed and fixed for 72 h at 4 °C in 4% paraformaldehyde. For neuropathological analysis, 40-μm-thick sagittal brain sections were obtained using a vibratome (Leica VT 1000S, Leica Biosystems, Buffalo Grove, IL) and stained with primary anti-microtubule-associated protein 2 monoclonal antibody (mAb; mouse anti-MAP2; 1:200; Sigma) or a mouse anti-synaptophysin mAb (1:50; Dako) for neuronal markers, or rabbit anti-ionized calcium-binding adaptor molecule 1 (Iba1) IgG (1:125; Wako) for microglia or rabbit anti-glial fibrillary acidic protein (GFAP) IgG (1:250; Dako) for astrocytes. Rhodamine-conjugated goat anti-mouse (1:50 Jackson ImmunoResearch) and Alexa Fluor 488-labeled goat anti-rabbit (1:200; Invitrogen) secondary antibodies (Abs) were employed to visualize primary Abs. Nuclei were counterstained with Hoechst 33342 (Invitrogen). Separate sections were incubated with mouse IgG1 (MOPC21, Sigma, M-9269) as primary isotype control or primary Ab was omitted. Immunolabeled brain slices were mounted on glass slides with fluorescence-protecting mounting medium (VectaShield, Vector laboratories, Burlingame, CA, cat# H1000) and overlaid by a coverslip.

Images were acquired using a Zeiss 200 M fluorescence deconvolution microscope equipped with a computer-controlled 3D stage and the appropriate filters for DAPI, FITC, CY3, and CY5 (Carl Zeiss Microscopy GmbH, Jena, Germany). Slidebook software (version 6, Intelligent Imaging Innovations, Inc., Denver, CO) was used for all image acquisition and analysis. The Z-stack images were deconvolved using a constrained iterative algorithm, and threshold segmentation was applied to estimate the percentage of the neuropil occupied by microtubule-associated protein 2 (MAP2^+^) neuronal dendrites and synaptophysin (SYP^+^) presynaptic terminals and compared between the different genotypes. For direct quantitative analysis of fluorescence intensities of GFAP in the frontal cerebral cortex or the hippocampus (CA1), we recorded 2D images and the sum of fluorescence intensities (SFLI) was quantified using the area of interest. The SFLI values were normalized to the controls for the measured area and adjusted for background by subtracting values obtained from sections incubated only with secondary antibodies. For quantification of Iba1^+^ microglia, cell bodies were counted in the cerebral cortex and hippocampus (CA1) on one side of three sagittal brain sections spaced 320 μm apart medial to lateral. The total cell bodies counts were normalized to the area.

### RNA isolation and quantitative RT-PCR

Total RNA from mice hippocampus was extracted and qRT-PCR was performed and analyzed as described previously with minor modifications [15, 16, 18]. Briefly, RNA was extracted using a RNeasy Mini kit (Qiagen, Valencia, CA, cat# 74104), according to the manufacturer’s instructions. The RNA quality and quantity for all the samples were determined using a NanoDrop^TM^ DS-11 spectrophotometer (De Novix Inc., USA). Purified RNA (500 ng) was used to generate a cDNA template. qRT-PCR was carried out using 10 μL of Power PCR SYBR green master mix (Applied Biosystems, Thermo Fisher Scientific, Life Technologies LTD., Warrington, UK), 1 μL of cDNA, 8 μL of PCR grade water, and 0.5 μL of PCR primer pair (primer concentration: 20 μM except CXCL10: 10 μM). The primer sequences used for amplification are listed in Table 1. The RT-PCR was performed in a QuantStudio™ 6 flex system Real-Time PCR System (Applied Biosystems/Life Technologies) using the following settings: 95 °C for 10 min, 95 °C for 30 s, 59 °C for 1 min, 72 °C for 1 min, and for 40 cycles and denaturation steps were added at the end of the amplification reaction for Tm analysis. RNA samples corresponding to three to four biological replicates were analyzed. The signal for internal control (glyceraldehyde-3-phosphate dehydrogenase, GAPDH) was used to normalize the data. The relative amount of mRNA of every gene normalized to GAPDH was calculated following the 2^−ΔΔCt^ method.

**Table 1:** Primers for qRT-PCR

Gene	Genebank	Primer sequence (5′-3′)
HIV-1 gp120	M19921	Fwd: TGAGCCAATTCCCATACATTATTG
		Rev: CCTGTTCCATTGAACGTCTTATTATTAC
mCcl2	NM_011333.3	Fwd: CCCAATGAGTAGGCTGGAGA
		Rev: TCTGGACCCATTCCTTCTTG
mCcl5/Rantes	NM_013653.3	Fwd: ACACCACTCCCTGCTGCTTT
		Rev: TGCTGCTGGTGTAGAAATACTCCTT
mCcl3/Mip1α	NM_011337.2 15	Fwd: GCGCCATATGGAGCTGACA
		Rev: GATGAATTGGCGTGGAATCTTC
mCcl4	NM_013652.2	Fwd: AGGGTTCTCAGCACCAATGG
		Rev: AGCTGCCGGGAGGTGTAAG
mCcr5/CD195	NM_009917.5	Fwd: CGAAAACACATGGTCAAACG
		Rev: GTTCTCCTGTGGATCGGGTA 20
mCxcr4/CD184	NM_009911.3	Fwd: CTGGCTGAAAAGGCAGTCTATGT
		Rev: CGTCGGCAAAGATGAAGTCA
mSdf-1/mCxcl12	NM_001012477.2	Fwd: TGCCCCTGCCGGTTCT 16
		Rev: GAGTGTTGAGGATTTTCAGATGCTT
mMx1	NM_010846.1	Fwd: AGAGCAAGTCTTCTTCAAGGATCAC
		Rev: GTGGCCTTCCCATCTTCCA
mCxcl10	NM_021274.2	Fwd: GCCGTCATTTTCTGCCTCAT
		Rev: GGCCCGTCATCGATATGG
mCxcl11	NM_019594.1	Fwd: GGCTTCCTTATGTTCAAACAGGG
		Rev: GCCGTTACTCGGGTAAATTACA
Gapdh	NM_008084.2	Fwd: AGGTCGGTGTGAACGGATTTG
		Rev: TGTAGACCATGTAGTTGAGGTCA

### GABA glutamate and dopamine serotonin RT^2^ Profiler™ PCR array

The RNA extracted from mice hippocampus was used to run GABA/glutamate (GG) and dopamine/serotonin (DS) RT^2^ PCR arrays (Qiagen). Each sample was reverse transcribed using the RT^2^ First-Strand kit (Qiagen), mixed with RT^2^ qPCR Master Mix containing SYBR Green (Qiagen), and aliquoted (10 μL) into each well of the RT^2^ Profiler™ PCR Arrays (Qiagen). Each sample was used to assess the expression of 168 genes related to neurotransmission. Samples were analyzed in the mouse GG (PAMM-152) and DS (PAMM-158) neurotransmitter systems by RT^2^ Profiler™ PCR arrays following the supplier’s instructions (SABioscience/Qiagen). The arrays were run on a QuantStudio 6 flex Real-Time PCR System (Applied Biosystems by Thermo Fisher). The RT^2^ Profiler™ PCR Array Data Analysis software package (version 3.5, Qiagen) used 2^−(ΔΔCT)^-based fold change calculations [38] and a modified Student’s *t* test to compute two-tail, equal variance *P* values. Data were normalized to the housekeeping genes actin beta (*Act*β), glucuronidase beta (*Gus*β), heat shock protein 90 alpha (cytosolic) class B member 1 (*Hsp90ab1*), and Gapdh.

### Ingenuity Pathway Analysis

The results of the RT^2^ Profiler arrays were further interrogated using Ingenuity Pathway Analysis software (IPA; Ingenuity® Systems, www.ingenuity.com; build version: 486617 M; content version: 46901286; release date: 2018-11-21). The core analysis function was employed for identification of functional and biological gene networks, upstream regulators as described previously with some modifications [15, 16, 18]. The following settings were used: reference set, Ingenuity Knowledge Base (genes + endogenous chemicals), and relationship to include direct; optional analyses: My Pathways My List; Network Interaction and Causal, interaction network include endogenous chemical molecules per network (35) network per analysis (25), causal networks score using causal path only; node type: all; data sources: all; confidence: experimentally observed and high predicted; species: all; tissues and cell lines (adipocytes, astrocytes, immune cells, neurons, nervous system); mutation: all; fold change: − 1.1 to 1.1. Right-tailed Fisher’s exact test was performed for gene enrichment analysis.

### Western blot analysis

Mouse brain tissue lysates were prepared as previously described with modifications [15, 39]. Briefly, the dissected cerebral cortex or hippocampus was lysed on ice using 600 μL of lysis buffer (a mixture of 10 mL of 1 × RIPA buffer, 100 μL of phosphatase inhibitor (Calbiochem), and one tablet of complete protease inhibitor cocktail (Roche; Indianapolis, IN). The tissue samples were homogenized using an electric pestle and 3 mL syringe in 1.5 mL Eppendorf tubes. The lysed samples were cleared by centrifugation (14,000×*g*, 15 min) at 4 °C, and the supernatant was collected as lysate. Total protein concentrations were determined using the bicinchoninic acid (BCA) protein assay kit (Pierce/Thermo Fisher Scientific; Rockford, IL). Equal amount of protein samples (50 μg) was mixed with 4× LDS sample buffer (Life Technologies, CA, USA) and 10× reducing agents (Invitrogen; Carlsbad, CA) and heated for 5 min at 100 °C. The samples were then electrophoretically separated on SDS-PAGE gels (NUPAGE^TM^; Invitrogen) and subsequently electro-transferred to polyvinyl difluoride (PVDF) membranes (Invitrogen, USA). The following antibodies were employed: phospho-p38 (1°Ab- 1:1000; anti-rabbit 2°Ab- 1:5000) (Cell Signaling; 9212), total p38 (1°Ab- 1:2000; anti-rabbit 2°Ab- 1:25,000) (Cell Signaling; 9211S), phospho-ERK1 (1°Ab- 1:1000; anti-rabbit 2°Ab- 1:5000) (Cell Signaling; 9101S), total ERK1 (1°Ab- 1:2000; anti-rabbit 2°Ab- 1:5000) (Cell Signaling; 9102), active JNK (1°Ab- 1:1000; anti-rabbit 2°Ab- 1:3000) (Promega; V93A 20542917), total JNK (1°Ab- 1:1000; anti-rabbit 2°Ab- 1:3000) (Cell Signaling; 9252), phospho-STAT1 serine 727 (1°Ab- 1:1000; anti-rabbit 2°Ab- 1:3000) (Cell Signaling; D3B7), total STAT1 (1°Ab- 1:1000; anti-rabbit 2°Ab- 1:3000) (Cell Signaling, 14994S), and housekeeping genes β-tubulin (1°Ab- 1:2000; anti-mouse 2°Ab- 1:10,000) (Sigma T9026) or GAPDH (1°Ab- 1:20,000; anti-mouse 2°Ab- 1:25,000) (Ambion; 4300). The secondary antibodies anti-mouse-HRP (AP128P) and anti-rabbit-HRP (111-036-045) were purchased from Millipore Sigma and Jackson Immuno Research. Western blots were imaged with a Bio-Rad ChemiDoc system (Bio-Rad, Hercules, CA) and analyzed using ImageJ software (NIH). Densitometric measurements were normalized against housekeeping β-tubulin or GAPDH expression levels.

### In vitro neurotoxicity experiments

Neurotoxicity experiments were performed as described earlier [17, 32–34]. Recombinant gp120 of HIV-1 strain SF2 was obtained from NIH AIDS Research and Reference Reagent Program and was reconstituted in 0.1% bovine serum albumin (BSA) at 100× the final concentration. Controls received BSA vehicle alone (0.001% final concentration). The specific inhibitors of ERK kinase (MEK), PD98059, and p38 MAPK, SB203580, were obtained from Calbiochem (San Diego, CA). Kinase inhibitors were dissolved in dimethyl sulfoxide (DMSO) at 1000× final concentration (0.1, − 2 μM), and the p38 MAPK inhibitor was added to the cerebrocortical cell cultures for 15 min prior to treatment with 200 pM HIVgp120 or PD98059. After 24-h incubation, surviving neurons were quantified by immunostaining for the neuron-specific marker MAP-2, and apoptotic nuclei were identified morphologically after staining nuclei with the DNA dye Hoechst 33342 [32–34]. Neuronal survival was calculated from the percentage of neurons remaining after subtraction of those that had undergone apoptosis. Two to six independent experiments were performed for each treatment.

### Statistical analysis

Experimental results are presented in combined box-dot plots with the 25th and 75th percentiles. The middle line of the box shows the median, and the mean is indicated by a “+.” The whiskers show the full range of values. Values in Additional file 1, B and C, are shown as line graphs. GraphPad Prism 7.03 (GraphPad Software, Inc., CA, USA) was employed for the generation of graphs and a comparison of more than two experimental groups used ANOVA followed by Tukey’s honestly significant difference (HSD) post hoc test. For behavioral outcomes in Barnes maze probe test and novel object recognition, ANOVA analysis was performed followed by Fisher’s protected least significant difference post hoc test using StatView software package (version 5.0.1; SAS Institute, Cary, NC). For the RT^2^ Profiler™ PCR Array Data analysis, QIAGEN data analysis center was used for 2^–(ΔΔCT)^-based fold change calculations and a modified Student’s *t* test to compute two tails, equal variance *P* values (http://www.qiagen.com/geneglobe). For gene enrichment analysis right-tailed Fisher’s exact test was performed. Statistical significance was set at *P* ≤ 0.05.

## Results

### IFNAR1 and HIVgp120 affect memory in a sex-dependent fashion

Mice of the following four genotypes were behaviorally assessed at 11 to 14 months of age: (1) IFNAR1KO × gp120 (IFNAR1^-/-^gp120^+^), (2) HIVgp120 (IFNAR1^+/+^gp120^+^), (3) IFNAR1KO (IFNAR1^-/-^gp120^-^; control), and (4) WT (IFNAR1^+/+^gp120^-^; control).

**Fig. 1:**
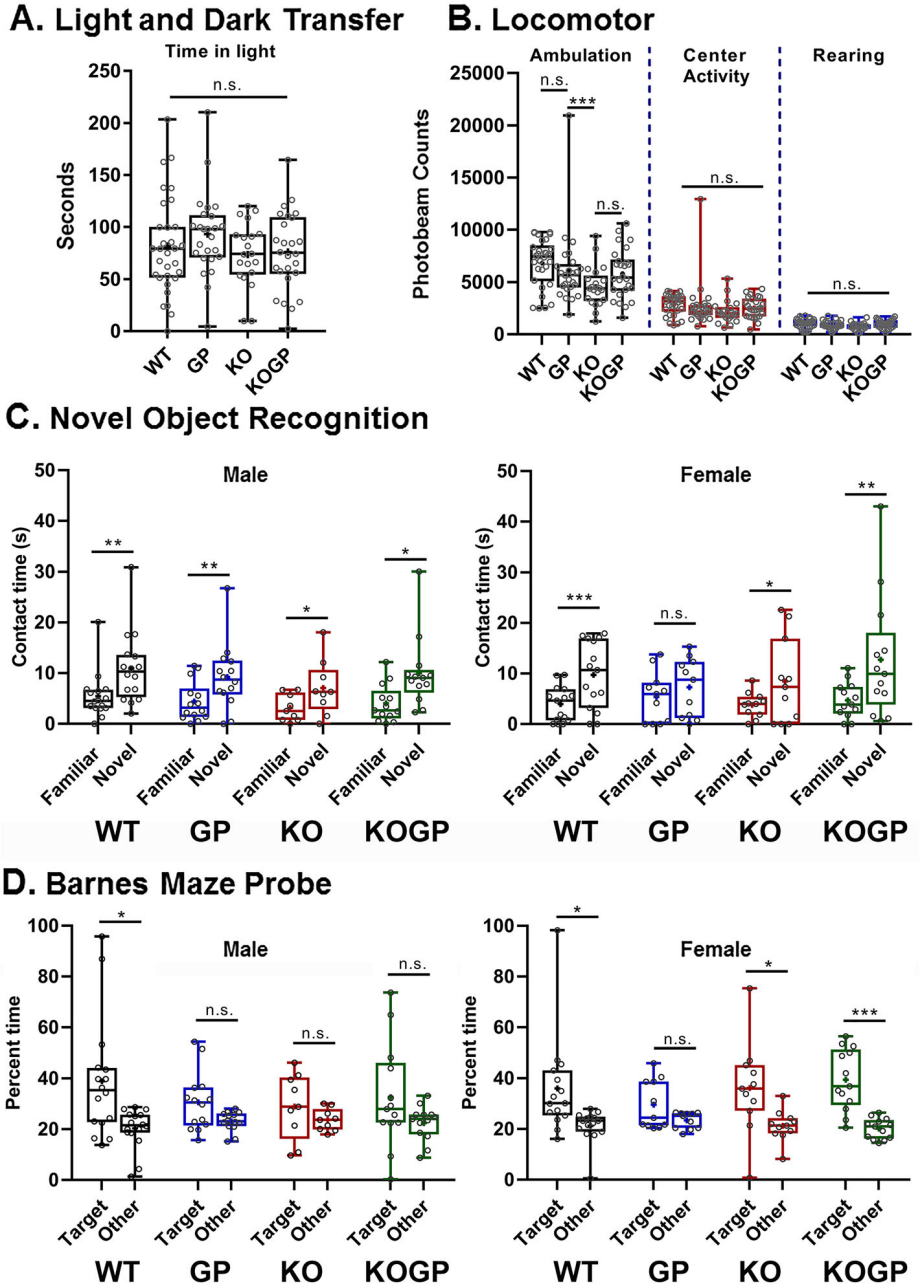
IFNAR1 deficiency rescues recognition and spatial memory of HIVgp120tg females. Eleven to 14-month-old mice were behaviorally assessed (genotype: *n* for males/*n* for females—WT (WT): 11/11, HIVgp120tg (GP): 9/8, IFNAR1KO (KO): 9/11, and IFNAR1KO × gp120 (KOGP): 13/14). Light and dark transfer test for anxiety-like behavior (**a**); Locomotor (ambulation, center activity, and rearing (**b**); novel object recognition (**c**; left panel: males, right panel females); Barnes maze probe test for spatial learning and memory (**d**; left panel: males, right panel females). Data is presented in combined box-dot plots with the 25th and 75th percentiles. The middle line of the box shows the median, and the mean is indicated by a “+.” Statistical analysis was performed as described in the methods section. ***P < 0.001, **P < 0.01, *P ≤ 0.05; ANOVAs Tukey’s HSD (**a**, **b**) and Fisher’s PLSD post hoc tests (**c**, **d**); *n* = 17–27 (males and females) per group/genotype (total *n* = 86 animals); n.s., not significant.

HIVgp120tg mice, but not WT, spent more time in the light compared to IFNAR1KO regardless of viral envelope expression (Fig. 1a). However, neither IFNAR1KO nor HIVgp120 expression, nor any interaction involving the two factors, affected the number of transitions into the light compartment. Time spent in the light compartment or dark-to-light transitions did not indicate any sex difference, regardless of genotype.

The locomotor test showed significant effects of time on each activity measure (counts went down across time as the mice habituated to the novel environment), but there were no significant interactions involving time, and therefore, the 2 h totals are shown in Fig. 1b. No sex differences or any interactions involving sex were detected. Significant effects of IFNAR1KO and significant IFNAR1KO × gp120 interactions, respectively, were found in ambulation (*P* < 0.05), but not on activity in the center of the cages and rearing (Fig. 1b). Also, all mice showed head tracking in the optomotor test, suggesting that vision was intact (Additional file 1A).

The novel object recognition test examined the difference between familiar vs. novel object contact time for each group separately. Males in all groups showed significantly more interest in the novel object than in the familiar object suggesting that recognition memory is not affected by IFNAR1KO and/or HIVgp120 expression. In contrast and revealing sexual dimorphism, female HIVgp120tg mice failed to distinguish familiar and novel objects, and this impairment was reversed by the IFNAR1KO (*P* < 0.01) (Fig. 1c).

The Barnes maze test indicated no significant effects of sex, nor any interaction involving sex, on the latency to escape and the number of errors made before escaping (Additional file 1B and C). While IFNAR1KO was associated with increased latencies to escape, there were no effects of either IFNAR1KO or HIVgp120 on errors made before escaping. In contrast, the probe test revealed differences in spatial memory based on sex and presence of IFNAR1 and HIVgp120. HIVgp120 compromised spatial memory in both sexes, but IFNARKO itself disrupted spatial memory in males while abrogating the impairment in females (Fig. 1d).

### IFNAR1KO partially prevents neuronal damage in HIVgp120tg mice

**Fig. 2:**
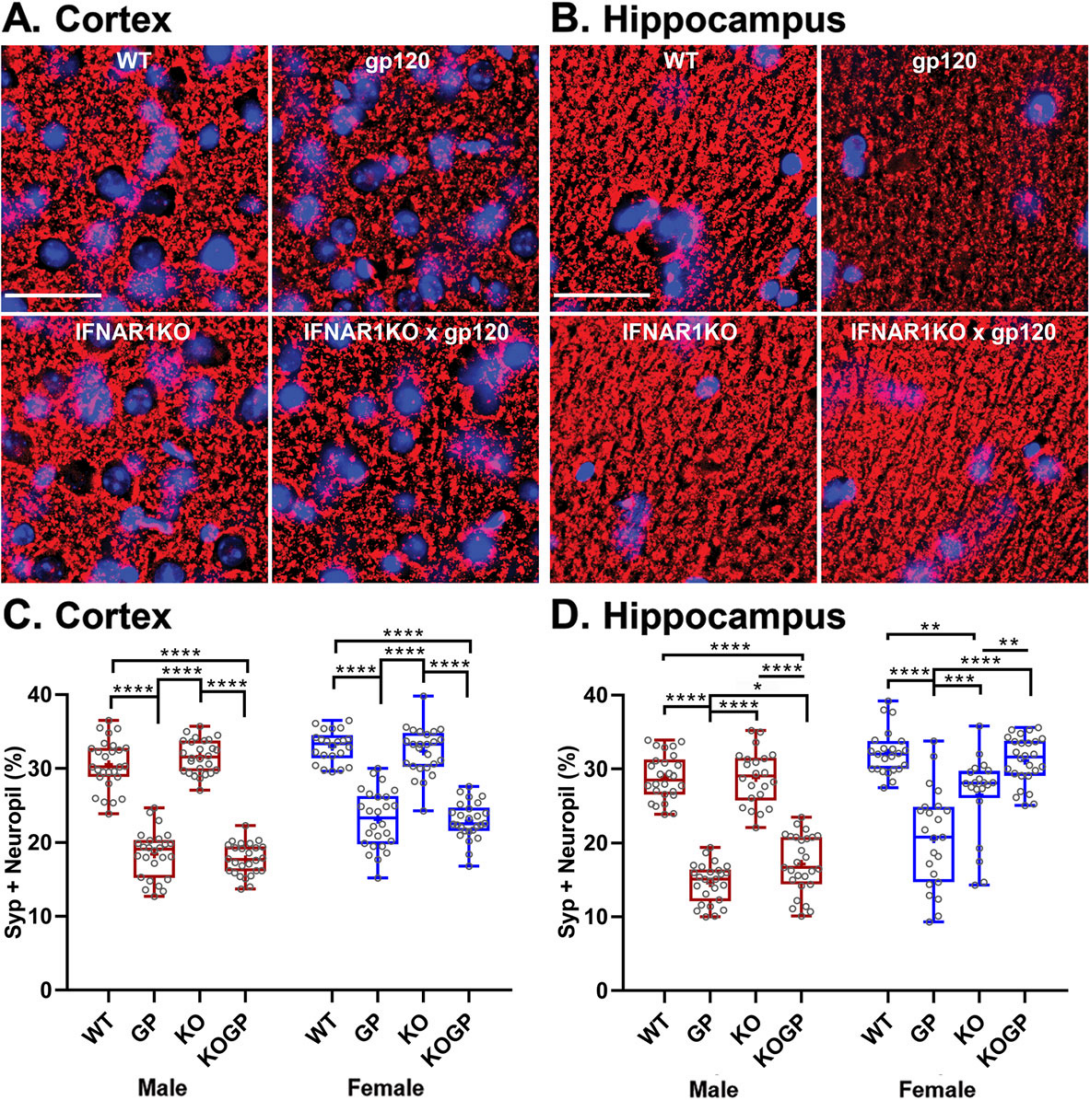
IFNAR1 deficiency partially ameliorates the loss of presynaptic terminals preferentially in the hippocampus of females. Representative images of the cortex (**a**; layer 3) and hippocampus (**b**; CA1) immunolabeled for neuronal synaptophysin (SYP); deconvolution microscopy; scale bar, 40 μm. **c**, **d** Quantification of microscopy data obtained in the cortex and hippocampus of sagittal brain sections of 12–14-month-old mice. Genotypes: WT (WT), HIVgp120tg (GP), IFNAR1KO (KO), and IFNAR1KO × gp120 (KOGP). Values are presented in combined box-dot plots with the 25th and 75th percentiles. The middle line of the box shows the median, and the mean is indicated by a “+”; ****P < 0.0001, ***P < 0.001, **P < 0.01, *P < 0.05; ANOVA and Tukey’s HSD post hoc test; *n* = 6 animals (3 males and 3 females) per group/genotype (total *n* = 24 animals)

Following behavioral assessment and thus at an age of 11–14 months, we analyzed the effect of IFNAR1-deficiency on presynaptic terminals and neuronal dendrites. First, we immunofluorescence-labeled sagittal brain sections for synaptophysin (SYP) in presynaptic terminals and for microtubule-associated protein 2 (MAP2) in neuronal dendrites, respectively. Quantitative analysis using deconvolution microscopy showed significant loss of SYP-positive neuropil (presynaptic terminals; *P* < 0.0001) in HIVgp120tg mice compared to WT in both frontal cortex (layer III; Fig. 2a, c) and hippocampus (CA1, stratum radiatum; Fig. 2b, d). IFNAR1KO did not affect presynaptic terminals in the cortex of both males and females. In the male hippocampus, there was no significant change between the WT and IFNAR1KO mice but the absence of IFNAR1 resulted in protection against the loss of SYP^+^ neuropil otherwise seen in HIVgp120 expressing mice (Fig. 2d, left panel). In contrast, in the female hippocampus, IFNAR1 deficiency resulted in a loss of SYP^+^ presynaptic terminals in the absence of HIVgp120 but paradoxically a more pronounced protection in IFNAR1KO × gp120 mice (Fig. 2d, right panel). Overall, the results indicated a protective effect of IFNAR1 deficiency against HIVgp120 induced loss for presynaptic terminals which was specific to the hippocampus and influenced by sex in that females were more protected than males.

**Fig. 3:**
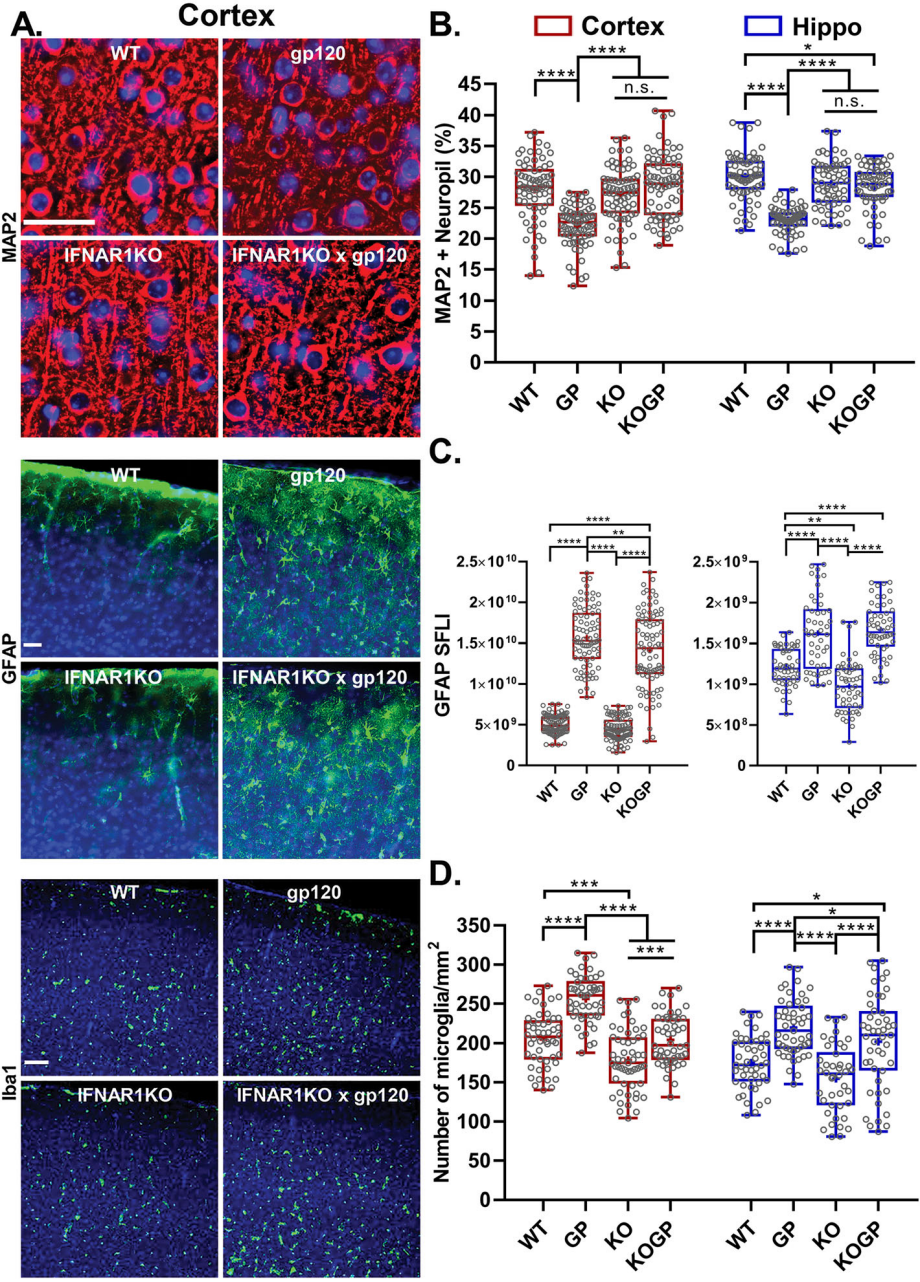
IFNAR1 deficiency prevents damage to neuronal dendrites and microgliosis but not astrocytosis in HIVgp120tg mice. Representative images of the frontal cerebral cortex (**a**) immunolabeled for neuronal MAP2 (MAP2; layer 3; deconvolution microscopy; scale bar, 40 μm); astrocytic GFAP (cortex, all layers; fluorescence microscopy; scale bar, 40 μm), and Iba^+^ microglia numbers (cortex, all layers; fluorescence microscopy; scale bar, 100 μm). **b**–**d** Quantification of microscopy data obtained in the cortex and hippocampus of sagittal brain sections of 12–14-month-old mice. Neuropil positive for neuronal MAP2 (**b**); Fluorescence signal for astrocytic GFAP (**c**); Counts of Iba1^+^ microglia (**d**). Genotypes: WT (WT), HIVgp120tg (GP), IFNAR1KO (KO), and IFNAR1KO × gp120 (KOGP). Values are presented in combined box-dot plots with the 25th and 75th percentiles. The middle line of the box shows the median, and the mean is indicated by a “+”; ****P < 0.0001, ***P < 0.001, **P < 0.01, *P < 0.05; ANOVA and Tukey’s HSD post hoc test; *n* = 6 animals (3 males and 3 females) per group/genotype (total *n* = 24 animals)

The analysis of MAP2^+^ neurites showed a significant reduction in HIVgp120tg mice in comparison to all the other three genotypes (*P* < 0.0001; Fig. 3a, b). The IFNAR1KO rescued HIVgp120tg mice from the loss of neuronal dendrites in both the cortex and hippocampus, and this effect on MAP2 was sex-independent.

### IFNAR1 deficiency regulates microglia activation but not astrocytosis

Quantitative immunofluorescence microscopy of immunofluorescence-labeled GFAP of astrocytes showed an increase in association with HIVgp120 expression regardless of IFNAR1 genotype (Fig. 3a middle panels, GFAP in the cortex, Fig. 3c quantification for the cortex and hippocampus). In the cortex, IFNAR1KO × gp120 mice showed a significant decrease (*P* < 0.01) in the expression of GFAP compared to HIVgp120tg mice, while in the hippocampus GFAP expression was unaffected by IFNAR1 deficiency in the HIVgp120tg mice. However, GFAP immunoreactivity in the hippocampus was decreased in IFNAR1KO compared to the WT mice (*P* < 0.01) suggesting that IFNAR1 is necessary to maintain baseline levels of the astrocytic protein GFAP in this region of the brain. No sex-dependent differences were observed for GFAP and astrocytosis.

Iba1 was used as the marker for labeling microglia, and positive cell bodies were counted in the cerebral cortex (layer III) and hippocampus (CA1) on one side of three sagittal brain sections spaced 320 μm apart medial to lateral for each animal. Iba1^+^ microglia counts were significantly higher in HIVgp120tg mice compared to the other three genotypes in both cortex and hippocampus (Fig. 3a bottom panels, Iba1 in the cortex, Fig. 3d quantification for the cortex and hippocampus; *P* < 0.05–0.0001). Similar to the astrocyte data, no sex differences were observed. IFNAR1KO mice had lower counts of Iba1^+^ cells compared to all other genotypes in both the cortex and hippocampus (*P* < 0.05–0.0001). In the cortex, IFNAR1KO × gp120 mice had counts of microglia similar to WT mice and significantly less than the HIVgp120tg mice (*P* < 0.0001). In the hippocampus, IFNAR1KO × gp120 mice showed a significant increase in the number of microglia compared to WT (*P* < 0.05) and IFNAR1KO (*P* < 0.0001) mice, but still, the counts were lower than in HIVgp120tg mice (*P* < 0.05) (Fig. 3d). Thus, IFNAR1 deficiency appeared to overall dampen microgliosis.

### IFNAR1KO affects the expression of the viral transgene and host genes

**Fig. 4:**
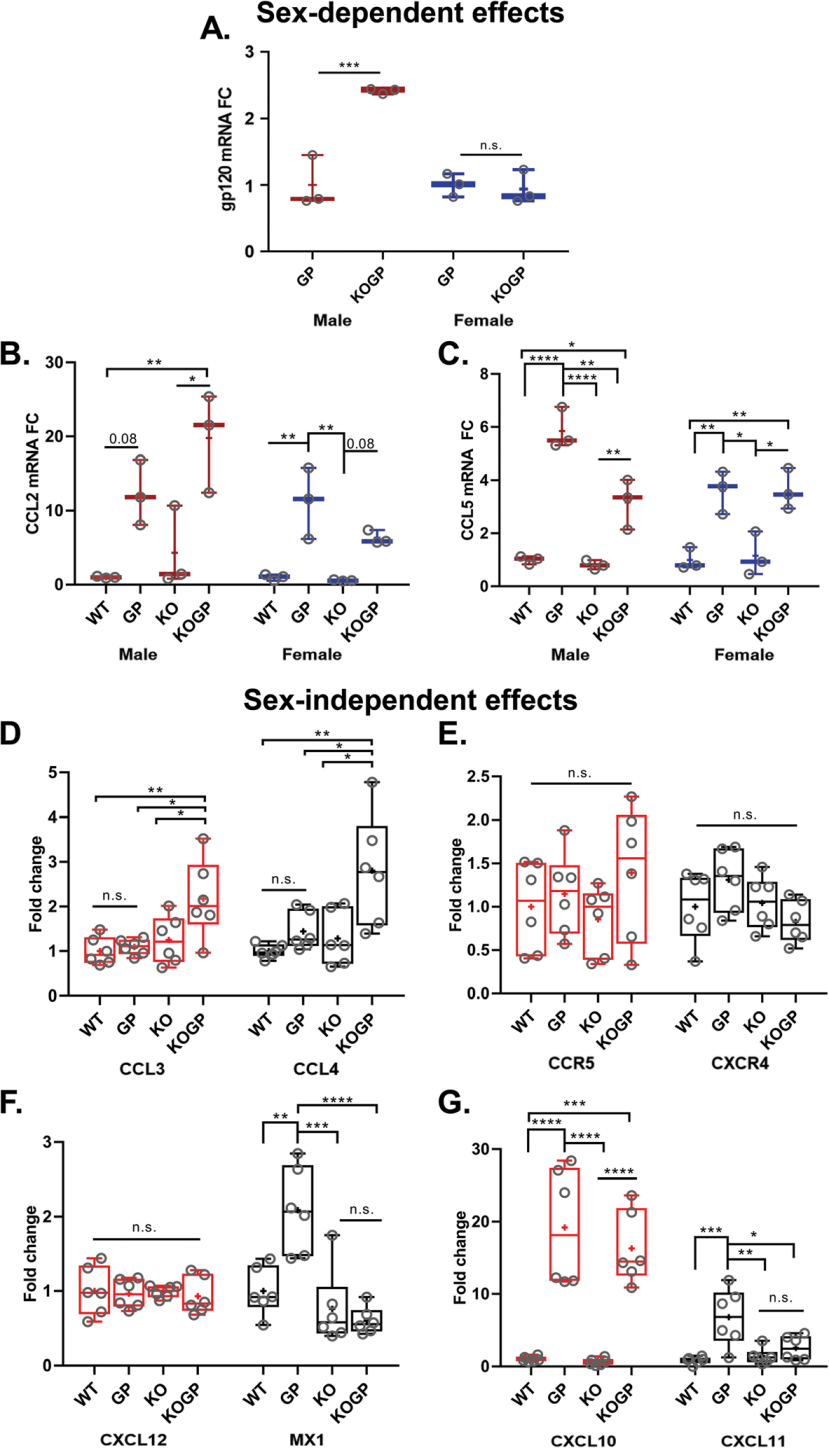
Sex-dependent and independent effects of IFNAR1 deficiency on RNA expression of viral HIVgp120 and host genes. The mRNA expression in the hippocampus of 11-14 months-old mice, were detected by quantitative RT-PCR as described in the material and method section. HIVgp120 (**a**); CCL2 (**b**) and CCL5 (**c**), CCL3 and CCL4 (**d**); CCR5 and CXCR4 (**e**); CXCL12 and MX1 (**f**); CXCL10 and CXCL11 (**g**). The obtained CT values were normalized to the level of GAPDH mRNA. Genotypes: WT (WT), HIVgp120tg (GP), IFNAR1KO (KO), and IFNAR1KO × gp120 (KOGP). Values are presented in combined box-dot plots with the 25th and 75th percentiles. The middle line of the box shows the median, and the mean is indicated by a “+”; ****P < 0.0001, ***P < 0.001, **P < 0.01, *P < 0.05; ANOVA and Tukey’s HSD post hoc test; *n* = 3 per sex/experimental group/genotype or 6 animals (3 males and 3 females combined) per group/genotype (total *n* = 24 animals); n.s., not significant

We next investigated if the neuroprotection conveyed by IFNAR1 deficiency in the hippocampus was due to a change in the expression of the viral envelope protein HIVgp120. The analysis by qRT-PCR revealed that IFNAR1KO results in differential RNA expression for HIVgp120 in males but not females (Fig. 4a). Expression of HIVgp120 was 2.5-fold higher in IFNAR1KO × gp120 males compared to their respective HIVgp120tg controls (*P* < 0.001).

We observed earlier that HIVgp120 expression in the brain induces ISGs, cytokines, and chemokines [15, 16]. In order to deduce the potential role of IFNAR1 in this innate immune response, we analyzed the effect of HIVgp120 and IFNAR1 deficiency on cytokine mRNA expression in the hippocampus. The results of qRT-PCR showed that the expression of CCL2 and CCL5 was significantly upregulated in HIVgp120tg compared to WT mice (*P* < 0.01 for CCL2 in females only, and *P* < 0.0001 and 0.01 in males and females, respectively, for CCL5). In males, CCL2 expression was highest for IFNAR1KO × gp120 mice and significantly increased compared to WT and IFNAR1KO mice (*P* < 0.05 and 0.01). In contrast, CCL5 expression in males was significantly increased in IFNAR1KO × gp120 mice compared to WT and IFNAR1KO controls (*P* < 0.05), but less than in HIVgp120tg mice (*P* < 0.01; Fig. 4c, left panel). In females, CCL5 expression was increased due to HIVgp120 irrespective of IFNAR1 (*P* < 0.05; Fig. 4c, right panel). CCL3 and CCL4 were not significantly upregulated in HIVgp120tg mice compared to the WT mice, but the knockout of IFNAR1 resulted in significant increases in IFNAR1KO × gp120 mice compared to the other three genotypes (Fig. 4d). Sex differences were found only for CCL2 and CCL5, but not CCL3 and CCL4.

The chemokine receptors CCR5 and CXCR4 are the co-receptors for HIV infections alongside its primary receptor CD4 [40, 41]. The qRT-PCR indicated that the mRNA expression of CCR5 and CXCR4 is not significantly different between the four genotypes (Fig. 4e). We also assayed CXCL12 mRNA expression because this natural ligand could compete with viral HIVgp120 for the interaction with CXCR4 [15, 40]. However, no significant effect was detected of HIVgp120 or IFNAR1 deficiency on the expression level of CXCL12 (Fig. 4f, left panel).

Expression of MX1, an interferon-induced GTP-binding protein with activity against DNA and RNA viruses [42], was significantly upregulated in the presence of HIVgp120 (*P* < 0.01; Fig. 4f, right panel). This upregulation of MX1 was absent in IFNAR1KO and IFNAR1KO × gp120 mice confirming the blockade of type I IFN signaling.

The chemokines CXCL10 and CXCL11 were assessed because we found their levels to be elevated in the brain of HIVgp120tg mice and CXCL10 has been linked to neuronal dysfunction and found to be overexpressed in the CNS of HIV encephalitis (HIVE) patients [8, 15, 16]. Results confirmed that the RNA expression of both chemokines was significantly upregulated in the presence of HIVgp120 (*P* < 0.0001 and *P* < 0.001, respectively; Fig. 4g). The upregulation of CXCL10 mRNA in the presence of HIVgp120 occurred regardless of the IFNAR1 genotype (Fig. 4g, left panel). In contrast, CXCL11 expression was significantly decreased in IFNAR1KO × gp120 compared to the HIVgp120tg mice hippocampus (*P* < 0.05; Fig. 4g, right panel). IFNAR1KO did not significantly affect the expression of CXCL10 and CXCL11 in the absence of HIVgp120.

### Sex-dependent effects of IFNAR1KO and HIVgp120 on neurotransmission-related gene networks

**Fig. 5:**
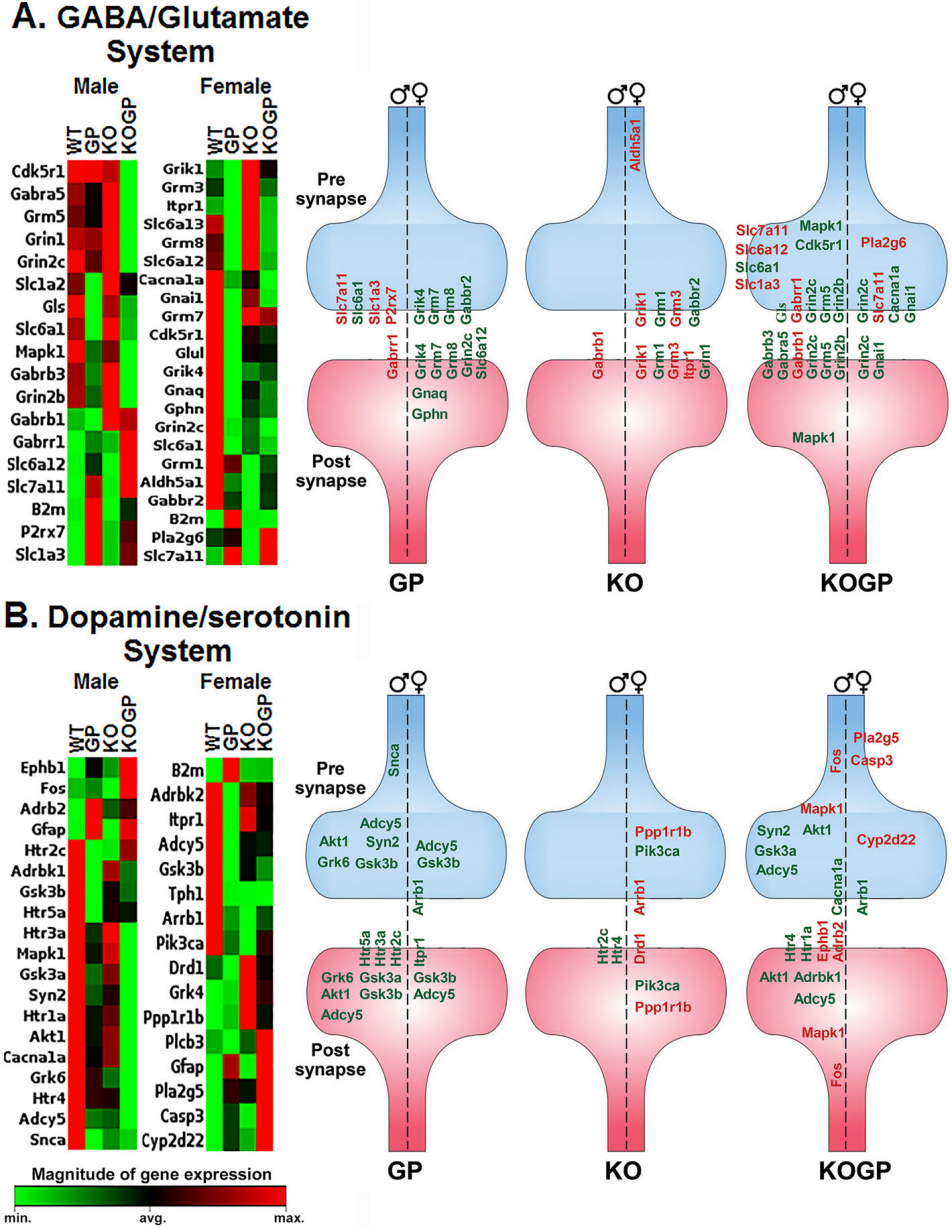
HIVgp120 and IFNAR1 cause sex-dependent, differential expression of genes related to neurotransmission systems in the hippocampus. Clustergram heat map of GABA/glutamate system (**a**) and dopamine/serotonin system (**b**) showing gene expression profile of WT, HIVgp120tg, IFNAR1-deficient mice with or without HIVgp120. Red indicated the higher gene expression while green indicates the lower gene expression in the sample set. Hippocampus RNA was analyzed using RT^2^ Profiler PCR Array and the associated Qiagen data analysis software. The heat maps represent significantly changed genes as the averages of three biological replicates. The schematic figure to the right represents pre- and post-synaptic distribution of the differentially expressed genes in neurons of each genotype. Genotypes: WT (WT), HIVgp120tg (GP), IFNAR1KO (KO), and IFNAR1KO × gp120 (KOGP); *n* = 6 animals (3 males and 3 females) per group/genotype (total *n* = 24 animals)

We recently showed that GABAergic, glutamatergic, dopaminergic, and serotoninergic neurotransmission systems all are altered at the RNA level in HIVgp120tg mice [15, 18]. To test whether IFNAR1KO ameliorated the disturbance of neurotransmission, we used RT^2^ PCR arrays to analyze the expression of 168 genes associated with neurotransmission by GABA and glutamate (GG array: 84 genes) and dopamine and serotonin (DS array: 84 genes) in the hippocampus. Compared to WT all other genotypes displayed significant sex-dependent changes in the expression of genes related to neurotransmission. The differentially expressed genes provided evidence for sex-dependent alterations of pre- and post-synaptic components and related signaling factors in HIVgp120tg mice with and without IFNAR1, and IFNAR1-deficient females (Fig. 5 and Table 2). IFNAR1KO males only displayed alterations in RNA for subunits of postsynaptic neurotransmitter receptors. Notably, HIVgp120 reduced expression of MAPK1/extracellularly regulated kinase (ERK), which has been implicated in HAND [43, 44], and is a major upstream regulator of cAMP-response element-binding protein (CREB) which in turn affects multiple components of neurotransmission.

**Table 2:** Genes differentially regulated in males and females in the GABA/glutamate and dopamine/serotonin neurotransmission systems

**GABAergic & glutamatergic neurotransmission systems (male)**								
**HIVgp120**			**IFNAR1KO**			**IFNAR1KO gp120**		
**Gene**	**Fold regulation**	***P*value**	**Gene**	**Fold regulation**	***P*value**	**Gene**	**Fold Regulation**	***P*value**
Gabrr1	4.00	0.041	Gabrb1	1.16	0.003	Cdk5r1	−1.21	0.020
P2rx7	1.46	0.002				Gabra5	−1.32	0.040
Slc1a2	−1.17	0.044				Gabrb1	1.13	0.046
Slc1a3	1.21	0.003				Gabrb3	−1.14	0.035
Slc6a1	−1.21	0.023				Gabrr1	13.52	0.006
Slc7a11	2.52	0.000				Gls	−1.15	0.027
B2m	1.90	0.017				Grin1	−1.29	0.027
						Grin2b	−1.35	0.036
						Grin2c	−1.91	0.041
						Grm5	−1.21	0.026
						Mapk1	−1.24	0.026
						Slc1a3	1.15	0.033
						Slc6a1	−1.18	0.045
						Slc6a12	3.50	0.024
						Slc7a11	2.77	0.000
**GABAergic & glutamatergic neurotransmission systems (female)**								
**HIVgp120**			**IFNAR1KO**			**IFNAR1KO gp120**		
**Gene**	**Fold regulation**	***P*value**	**Gene**	**Fold regulation**	***P*value**	**Gene**	**Fold regulation**	***P*value**
Cdk5r1	−1.25	0.047	Aldh5a1	−1.30	0.039	Cacna1a	−1.60	0.011
Gabbr2	−1.36	0.031	Gabbr2	−1.74	0.005	Gnai1	−1.26	0.010
Glul	−1.39	0.014	Grik1	1.36	0.040	Grin2c	−1.77	0.029
Gnai1	−1.27	0.046	Grm1	−1.47	0.030	Pla2g6	1.16	0.015
Gnaq	−1.18	0.005	Grm3	1.21	0.015	Slc7a11	1.85	0.044
Gphn	−1.38	0.025	Itpr1	1.16	0.043			
Grik4	−1.66	0.048						
Grin2c	−1.71	0.030						
Grm7	−1.25	0.021						
Grm8	−1.36	0.032						
Slc6a1	−1.33	0.023						
Slc6a12	−2.50	0.039						
Slc6a13	−2.11	0.017						
Slc7a11	1.84	0.002						
B2m	1.73	0.024						
**Dopaminergic & serotonergic neurotransmission systems (male)**								
**HIVgp120**			**IFNAR1KO**			**IFNAR1KO gp120**		
**Gene**	**Fold regulation**	***P*value**	**Gene**	**Fold regulation**	***P*value**	**Gene**	**Fold regulation**	***P*value**
Adcy5	−1.27	0.018	Htr2c	−1.27	0.0449	Adcy5	−1.42	0.002
Adrbk1	−1.21	0.045	Htr4	−1.17	0.0391	Adrb2	1.39	0.009
Akt1	−1.14	0.014				Adrbk1	−1.14	0.008
Gfap	14.54	0.000				Akt1	−1.30	0.001
Grk6	−1.15	0.036				Cacna1a	−1.63	0.022
Gsk3a	−1.18	0.023				Ephb1	1.77	0.004
Gsk3b	−1.18	0.029				Fos	1.90	0.014
Htr2c	−1.29	0.023				Gfap	13.82	0.000
Htr3a	−1.21	0.027				Grk6	−1.46	0.003
Htr5a	−1.55	0.032				Gsk3a	−1.27	0.006
Snca	−1.25	0.037				Htr1a	−1.65	0.024
Syn2	−1.20	0.047				Htr4	−1.49	0.008
						Mapk1	−1.27	0.046
						Syn2	−1.34	0.018
**Dopaminergic & serotonergic neurotransmission systems (female)**								
**HIVgp120**			**IFNAR1KO**			**IFNAR1KO gp120**		
**Gene**	**Fold regulation**	***P*value**	**Gene**	**Fold regulation**	***P*value**	**Gene**	**Fold regulation**	***P*value**
Adcy5	−1.45	0.020	Arrb1	−1.59	0.018	Arrb1	−1.32	0.033
Adrbk2	−1.26	0.010	Drd1	1.56	0.046	Casp3	1.23	0.024
Arrb1	−1.43	0.014	Grk4	1.45	0.046	Cyp2d22	1.34	0.050
Gfap	10.43	0.001	Pik3ca	−1.37	0.045	Gfap	12.41	0.000
Gsk3b	−1.26	0.016	Ppp1r1b	1.51	0.048	Gsk3b	−1.19	0.036
Itpr1	−1.13	0.040				Pla2g5	4.07	0.025
Tph1	−2.24	0.047				Plcb3	1.31	0.033
B2m	1.89	0.012						

**Fig. 6:**
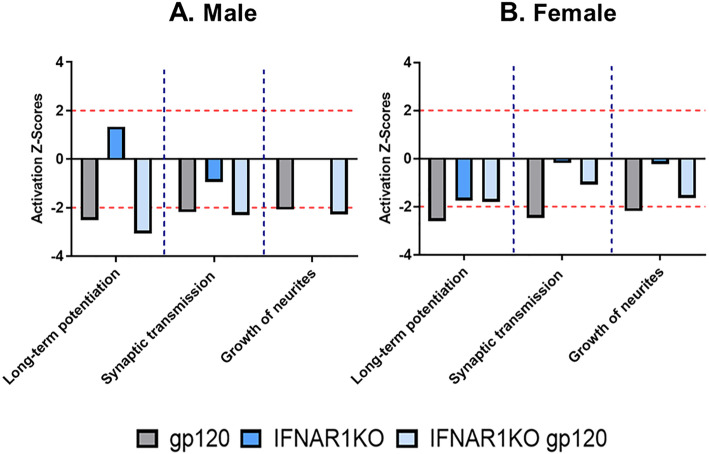
IFNAR1KO modifies the activity of functional, biological pathways affected by HIVgp120. Gene expression data obtained from RT^2^ Profiler PCR Arrays were interrogated using Ingenuity Pathway Analysis (IPA) software to analyze neurotransmission-related biological pathways affected by HIVgp120 and IFNAR1 deficiency. Note that most pathways were predicted to be reduced in activity except for long-term potentiation in IFNAR1KO males. The dashed lines at 2 and − 2 on the axis for activation *Z*-score indicate the significance threshold for a predicted effect on the activity of the given pathway (*P < 0.05); *n* = 6 animals (3 males (**a**) and 3 females (**b**) per group/genotype (total *n* = 24 animals)

For further bioinformatics analysis, the data were uploaded into the Ingenuity Pathway Analysis (IPA) software application (Qiagen). The core analysis function in IPA was used to interrogate the data in the context of biological processes, canonical pathways, and networks. IPA predicted the diminished activation of several functional gene networks in both males and females of HIVgp120tg mice: (i) reduced long-term potentiation (*z*-score = − 2.51 (M), − 2.59 (F)), (ii) synaptic transmission of nervous tissue (*z*-score = − 2.18 (M), − 2.47 (F)), and (iii) growth of neurites (*z*-score = − 2.9 (M), − 2.18 (F)). For IFNAR1KO × gp120 mice IPA indicated a slight mitigation of the HIVgp120-associated effects in females but not in males (Fig. 6a, b). In IFNAR1KO mice IPA showed increased long-term potentiation in males but the reduction in females, with a decline of overall synaptic transmission in males.

IPA also deduced several upstream regulators affected by HIVgp120 that explain the observed expression changes [45], such as for IFNAR1KO × gp120 males and HIVgp120tg females an inhibition of CREB binding protein (CREBBP; *z*-score = − 2.326 and − 2.00, respectively). The IFNAR1KO alone was not predicted to affect CREBBP in females. However, IPA implicated huntingtin (HTT) as an activated upstream regulator (*z*-score = 2.129) in HIVgp120tg females. The complete list of upstream regulators and their *z*-score values are shown in Table 3.

**Table 3:** Ingenuity Pathway Analysis (IPA) predicted upstream regulators in male vs female

	Upstream regulator	Molecule type	Predicted activation state	Activation *z*-score	*P* value
**Males**					
HIVgp120	None scored				
IFNAR1KO	None scored				
IFNAR1KOgp120	CREBBP	Transcription regulator	Inhibited	− 2.236	0.0000532
**Females**					
HIVgp120	HTT	Transcription regulator	Activated	2.129	2.3E−18
	CREBBP	Transcription regulator	Inhibited	− 2.0	0.0000145
IFNAR1KO	CREBBP	Transcription regulator	Inhibited	− 2.0	0.000054
IFNAR1KOgp120	CREBBP	Transcription regulator	Inhibited	− 2.0	0.0000209

**Fig. 7:**
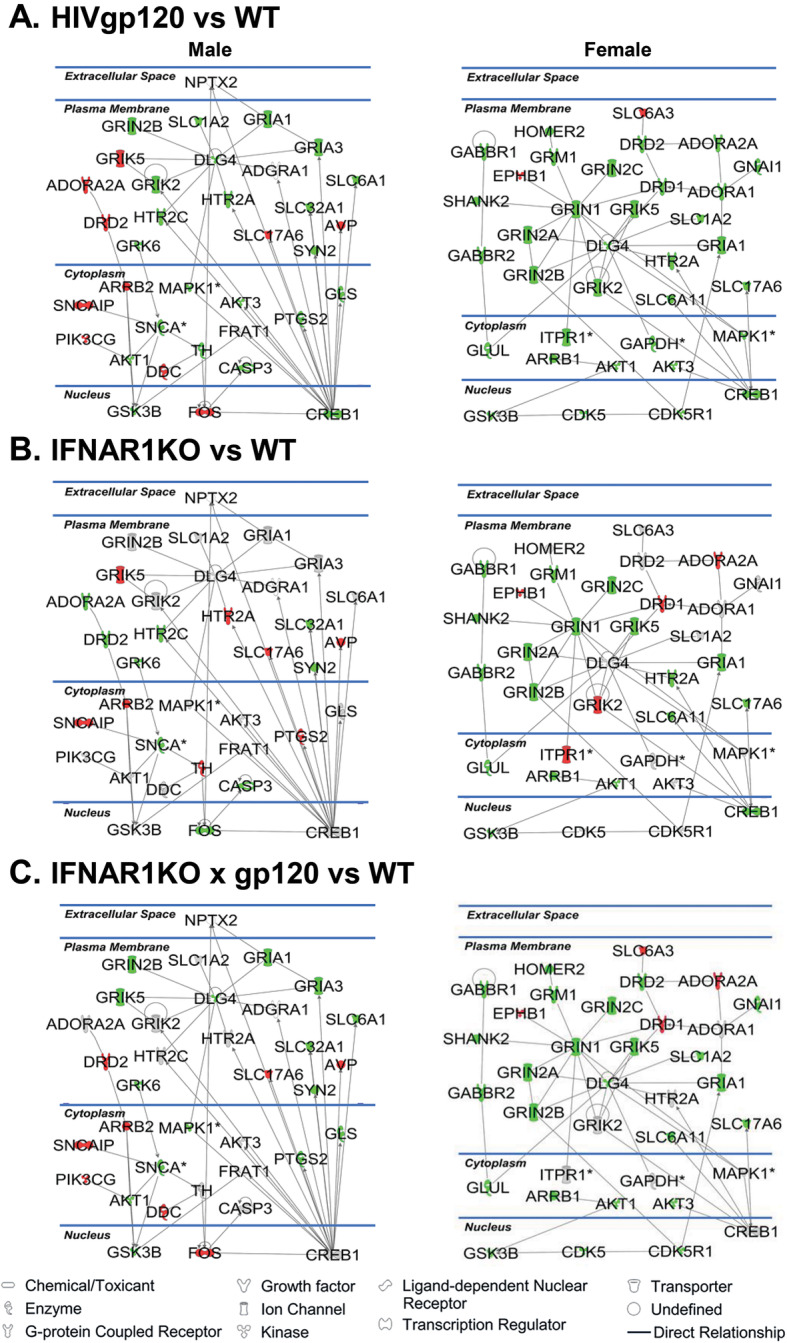
Functional neural gene networks of neurotransmission affected by HIVgp120 and IFNAR1 deficiency. RNA expression data obtained with the GABA/glutamate and dopamine/serotonin RT^2^ Profiler PCR Array were analyzed using IPA software. *Green* indicates downregulated while *red* reflects upregulated genes respectively. The components without color represent genes that were included by IPA without experimentally determined expression levels. IPA identified for each sex a different highest scoring gene direct interaction network in which alterations on expression levels were driven by HIVgp120 in the absence or presence of IFNAR1. The solid lines represent a direct relationship and the arrow indicates the direction of action. *Indicates genes for which differential regulation reached significance in the RT^2^ Profiler PCR Array (*P < 0.05; modified *t* test). RNA expression data were derived from *n* = 6 animals (3 males and 3 females) per group/genotype (total *n* = 24 animals)

IPA generated for males and females high scoring functional gene networks that were altered in the hippocampus of HIVgp120, IFNAR1KO, and IFNAR1KO × gp120 in comparison to the WT. The high scoring network for HIVgp120tg males (score: 57; focus molecules: 32; Table 4) is implicated in behavior, neurological disease, and cell to cell signaling interaction. The network for HIVgp120tg females (score: 66; focus molecule: 35; Table 4) is linked to cell-to-cell signaling, nervous system development, and behavior. These networks were affected by IFNAR1-deficiency as shown in Fig. 7. Notably, CREB1, an important regulator of neuronal pre-and postsynaptic function [44], was downregulated in both HIVgp120tg males and females (Fig. 7a) and IFNAR1KO females (Fig. 7b) while its expression was unaltered compared to WT control in IFNAR1KO × gp120 mice (Fig. 7c).

**Table 4:** List of molecules comprising the top-scoring network in male vs female identified by Ingenuity Pathway Analysis (IPA)

Sex	Molecules in network	Score	Focus molecules	Top diseases and functions
Males	ADGRA1,ADORA2A,AKT1,AKT3,ARRB2,AVP,CASP3,CREB1,DDC,DLG4,DRD2,FOS,FRAT1,GLS,GRIA1,GRIA3,GRIK2,GRIK5,GRIN2B,GRK6,GSK3B,HTR2A,HTR2C,MAPK1,NPTX2,PIK3CG,PTGS2,SLC17A6,SLC1A2,SLC32A1,SLC6A1,SNCA,SNCAIP,SYN2,TH	57	32	Behavior, cell-to-cell signaling and interaction, neurological disease
Females	ADORA1,ADORA2A,AKT1,AKT3,ARRB1,CDK5,CDK5R1,CREB1,DLG4,DRD1,DRD2,EPHB1,GABBR1,GABBR2,GAPDH,GLUL,GNAI1,GRIA1,GRIK2,GRIK5,GRIN1,GRIN2A,GRIN2B,GRIN2C,GRM1,GSK3B,HOMER2,HTR2A,ITPR1,MAPK1,SHANK2,SLC17A6,SLC1A2,SLC6A11,SLC6A3	66	35	Behavior, cell-to-cell signaling and interaction, nervous system development and function

### IFNAR1 contributes to HIVgp120 effects on MAPK activity

**Fig. 8:**
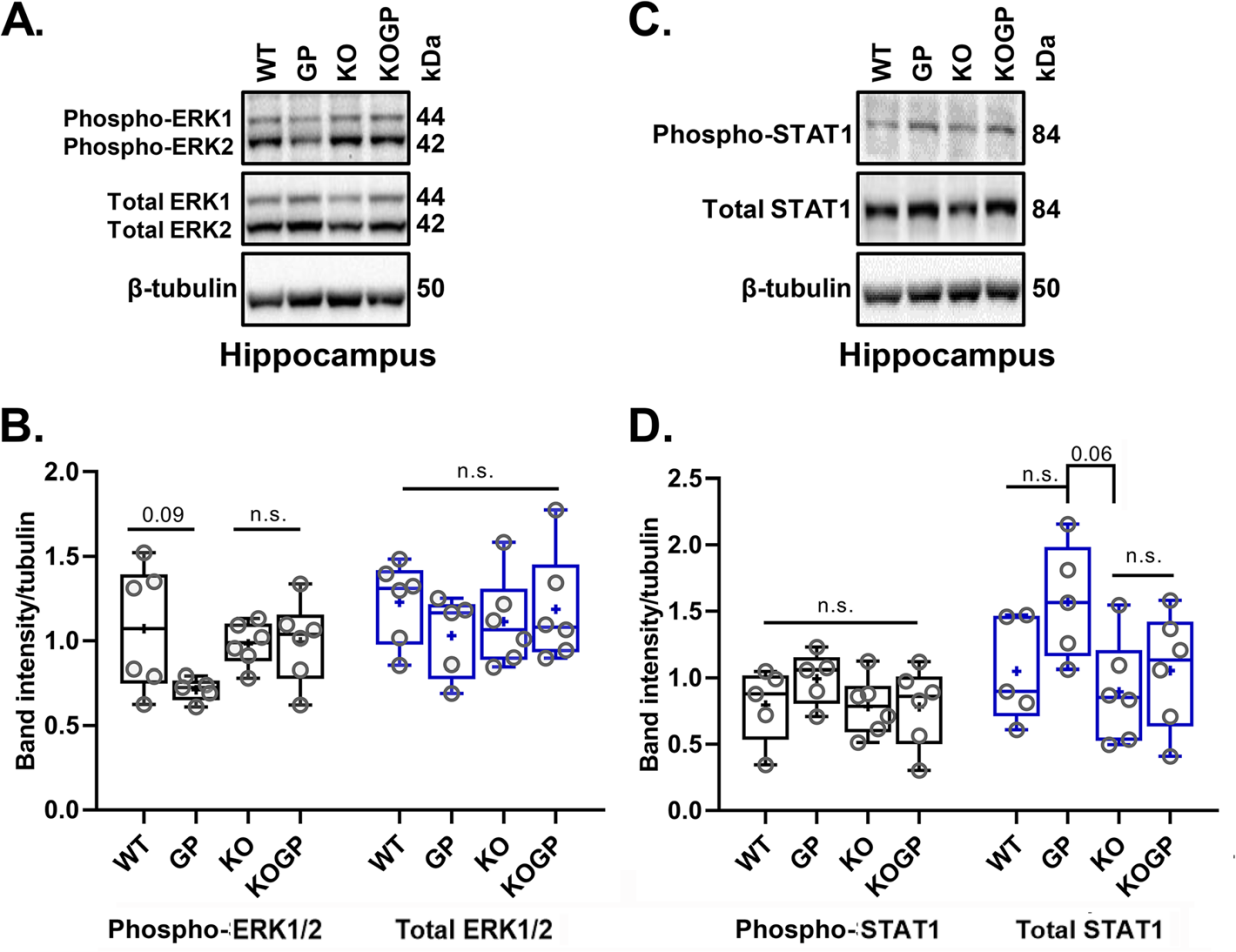
IFNAR1 deficiency abrogates activity loss of ERK1/2 and upregulation of STAT1 in the hippocampus of HIVgp120tg mice. The expression level of phospho-ERK1 and phospho-STAT1 was assessed by Western blot analysis. Representative Western blot of phospho-ERK1/2, ERK1/2, and β-tubulin in the hippocampus (**a**). The graph shows the quantification of phospho-ERK and ERK by densitometry using normalization to GAPDH (**b**). Representative Western blot of phospho-STAT1, STAT1, and β-tubulin in the hippocampus (**c**). The graph shows the densitometry analysis of phospho-STAT1 and STAT1 normalized to GAPDH (**d**). Genotypes: WT (WT), HIVgp120tg (GP), IFNAR1KO (KO), and IFNAR1KO × gp120 (KOGP). Values are presented in combined box-dot plots with the 25th and 75th percentiles. The middle line of the box shows the median, and the mean is indicated by a “+”; number on the bar indicates *P* value, *P* < 0.05 as cut-off for significance; n.s., not significant; ANOVA and Tukey’s HSD post hoc test; brain tissues were derived from *n* = 6 of 11–14-month-old animals (3 males and 3 females) per group/genotype (total *n* = 24 animals)

ERK1/2 is a major upstream regulator of CREB1 that has also been implicated in HAND [43, 44]. We found here that the hippocampus of HIVgp120tg mice displayed a reduction in phospho-ERK1/2 (p-ERK1/2) compared to WT controls (*P* = 0.091), and IFNAR1 deficiency abrogated this effect (Fig. 8a, b). In contrast to the RNA expression of neurotransmission-related genes, the Western blotting experiments using 6 mice per genotype (3 males and 3 females) showed no indication of sexual dimorphism.

Since IFNα/β bind IFNAR1/2 receptors and signal through the JAK/STAT pathway [25, 26], we also investigated the level of phospho-STAT1 (p-STAT1) and STAT1. Western blotting indicated that p-STAT1 was slightly elevated above the baseline level by HIVgp120 in the presence of IFNAR1 but without reaching significance. Expression of STAT1 protein was higher in the hippocampus of HIVgp120tg mice compared to the other genotypes with a reduction in IFNAR1KO (*P* = 0.06; Fig. 8c, d) confirming the functional disruption of IFNα/β signaling.

**Fig. 9:**
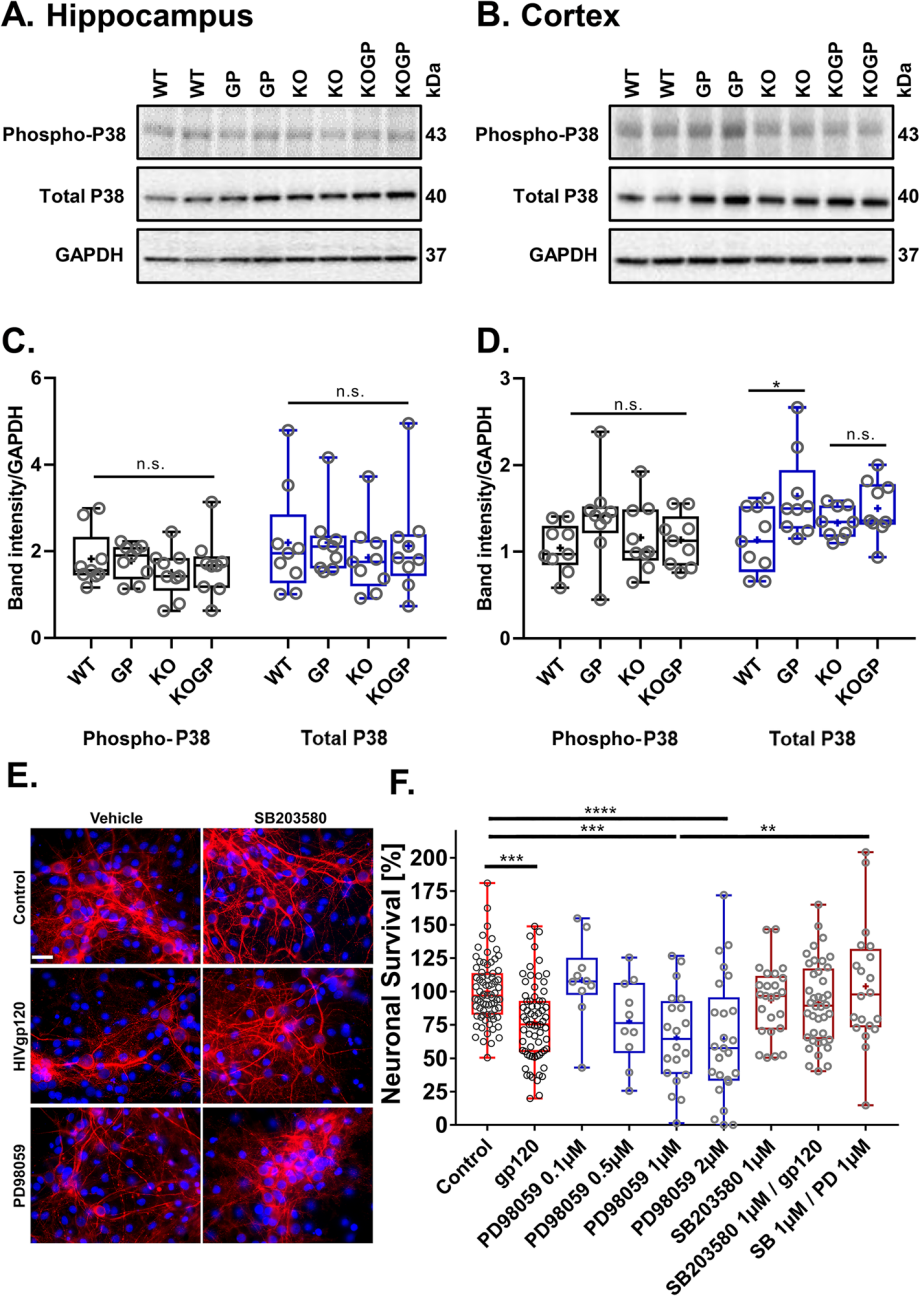
IFNAR1KO limits activation of p38 MAPK in the cerebral cortex of HIVgp120tg mice, and inhibition of the active kinase is neuroprotective. The protein levels of phospho-p38 (phospho-p38) and p38 MAPK protein (p38) were measured by Western blot analysis in the cerebral cortex (**a**) and hippocampus (**b**). Densitometric quantification of p-p38 and p38 in the cortex (**c**) and in the hippocampus (**d**). **e**, **f** Mixed neuronal-glial cerebrocortical cell cultures from rat were grown on glass coverslips and exposed between DIV 17–23 for 24 h to gp120 of HIV-1 (200 pM) or the ERK kinase inhibitor PD98059 (01, 0.5, 1, or 2 μM) in the presence or absence of the p38 MAPK blocker SB203580 (1 μM). Representative samples are shown in **e**, neuronal MAP-2 (red), nuclear DNA (blue). Subsequently, neuronal survival was assessed as described in methods (**f**). Genotypes: WT (WT), HIVgp120tg (GP), IFNAR1KO (KO), and IFNAR1KO × gp120 (KOGP). Values are presented in combined box-dot plots with the 25th and 75th percentiles. The middle line of the box shows the median, and the mean is indicated by a “+”; ****P < 0.0001, ***P < 0.001, **P < 0.01, *P < 0.05; ANOVA and Tukey’s HSD post hoc test; n.s., not significant; **a**–**d**, *n* = 9–10 of 11–14-month-old animals (each 4–5 males and 4–5 females) per group/genotype (total *n* = 37 animals). **f**, *n* = 2–6 independent experiments per condition; circles represent average neuronal survival observed per coverslip

Another regulator of CREB1 is the stress-activated p38 MAPK [44], which is also essential for neuronal death and microglia/macrophage activation in HIVgp120-induced neurotoxicity [32–34]. To quantify active p38 MAPK, we performed Western blots for the phosphorylated kinase and its total protein. Probing hippocampal and cerebrocortical tissues, we found that phospho-p38 MAPK was not significantly altered in any of the four tested genotypes; however, expression in the HIVgp120tg mice trended higher compared to WT (*P* < 0.21, Tukey HSD). The total-p38 (p38) protein levels were increased in the HIVgp120-expressing cortex (*P* < 0.05 for p38 protein; 10 mice per genotype, 5 males, and 5 females) but not hippocampus compared to WT control (Fig. 9a–d). The IFNAR1KO mice with or without HIVgp120 lacked a difference in phospho-p38 levels, suggesting IFNAR1 contributes to HIVgp120-induced phosphorylation of p38 MAPK. Overall, the data showed that IFNAR1 contributed to the effects of HIVgp120 on p38 MAPK.

Our previous studies have shown that neurons display the highest baseline levels of phosphorylated p38 MAPK in the mixed neuro-glial cell populations [17, 34]. In an in vitro approach using cerebrocortical cell cultures, we found that inhibition of ERK1/2 activation with increasing concentrations of the ERK kinase (MEK) inhibitor PD98059 caused neurotoxicity to a similar extent as HIVgp120 (Fig. 9e). Moreover, both the toxicity induced by the viral protein and the MEK inhibitor were completely abrogated by blocking the activity of p38 MAPK with SB203580. These findings were in line with the observations in the HIVgp120-expressing mouse brains and suggested that both the reduction of active ERK1/2 and the activity of p38 MAPK are mechanistic components of HIVgp120 neurotoxicity in the presence of IFNAR1.

In contrast to p38 MAPK, the stress-related cJun N-terminal kinase (JNK) did not display any significant changes in terms of phosphorylation or total protein level in the cortex and hippocampus of HIVgp120tg, IFNAR1KO, or IFNAR1KO × gp120 mice compared to WT controls, indicating that JNK activity was not significantly changed by chronic exposure to the viral envelope protein regardless of IFNAR1 (Additional file 2A-D).

## Discussion

HIV infection of the CNS often leads to brain injury, and HAND persists despite the use of cART, but the neuropathological mechanisms are still not completely understood [1, 46, 47]. However, infection with HIV triggers an IFN response [23], and IFNα/β and IFNα/γ all can inhibit HIV-1 infection in the periphery and in the brain [48–50]. We recently found that similar to SIV-infected rhesus macaques and mice intracerebrally injected with HIV-1 infected human macrophages, HIVgp120tg mice mount a type I IFN response in the CNS [15, 16, 27, 28, 30]. IFNα is produced by a wide variety of nucleated cells that exist in or can enter the CNS, including astrocytes, microglia, neurons, macrophages, and T-lymphocytes [28, 51]. IFNα is an effective anti-viral host factor [52, 53], but prolonged expression of IFNα causes cognitive dysfunction in the HIV-1 exposed CNS [28, 30, 54]. Conversely, IFNβ, which can be produced by parenchymal and immune cells [25, 26, 55], has been reported to control of HIV and SIV infection in the brain [27, 29], and we have shown that intranasal IFNβ treatment confers in vivo neuronal protection against toxicity of viral HIVgp120 [16]. These same treatment experiments indicated that endogenous levels of IFNβ were insufficient to provide neuroprotection over time and therefore raised the possibility that IFNAR1 signaling could contribute to CNS injury because of the presence of endogenous IFNα [16]. Indeed, the present study provides evidence that IFNAR1 contributes to HIVgp120-induced neuronal injury and neurocognitive and memory impairment. In addition, we find that the effect of IFNAR1 is, at least in part, sex-dependent.

Gender-specific studies in HIV-positive patients revealed that HIV-positive women display higher immune activation and have a higher risk of developing AIDS [56]. Additionally, a recent report of the Women’s Interagency HIV study found evidence that women maybe more vulnerable to cognitive impairment [57]. Murine models of HIV-brain injury show some data to support this point as female HIV-1 transgenic rats showed worse sustained attention than male rats [58]. While our behavioral assessment found that recognition memory was only impaired in female HIVgp120tg mice, it was rescued by IFNAR1 deficiency. Interestingly, not all memory types are affected in a sex-dependent manner as the Barnes maze paradigm revealed spatial learning and memory was impaired in both sexes of HIVgp120tg mice but the knockout of IFNAR1 rescued memory function in females while compromising it independently of HIVgp120tg in males. The Barnes maze probe test is sensitive to impaired hippocampal function [59], and this result fits well with the observation that pre-synaptic terminals in the hippocampus of males were diminished while the pre-synaptic terminals in the females of IFNAR1KO × gp120 mice were indistinguishable from WT control. Of note, the reduction of SYP^+^ presynaptic terminals in the female hippocampus and spatial memory impairment of male IFNAR1KO mice indicates an important role of the type I IFN receptor in normal brain function via mechanisms that warrant further investigation [55]. Others reported recently in a mouse model that lack of IFNβ signaling via IFNAR1 caused Lewy body formation, motor and cognitive learning impairments, reduction in dopaminergic neurons, defective dopamine signaling, and a Parkinson’s disease phenotype [60]. However, beyond the sex-dependent spatial memory impairment and reduced ambulatory locomotor activity, we did not observe a comparably severe phenotype in the absence of IFNAR1.

However, in HIVgp120tg mice, damage to MAP2^+^ neurites seems to be more dependent on IFNAR1 than injury to SYP^+^ presynaptic terminals, and effects on the latter are sex-dependent. Genetic ablation of IFNAR1 ameliorated the loss of MAP2^+^ neurites in the cortex and hippocampus with no difference between the sexes. In contrast, the protection of SYP^+^ presynaptic terminals was sex-dependent and specific to the brain region as it occurred only in the hippocampus. SYP^+^ presynaptic terminals in the cerebral cortex were more reduced in HIVgp120tg males than females but unaffected by IFNAR1 deficiency in both sexes. In contrast, presynaptic terminals were more protected in female than male hippocampus of IFNAR1KO × gp120 brains. This latter effect may be explained, at least in part, by higher HIVgp120 mRNA expression in the hippocampus of IFNAR1-deficient males compared to females. Thus, knocking out IFNAR1 in HIVgp120tg mice revealed that injury to neurites and presynaptic terminals occurred through different mechanisms, one dependent, and the other largely independent of IFNAR1. Moreover, the involvement of IFNAR1 in damage to SYP^+^ presynaptic terminals is dependent on brain region and sex.

The histopathological hallmarks of HIV-1-induced neuropathology include microgliosis and astrocytosis [12, 14] and are recapitulated in HIVgp120tg mice [15, 16, 19]. IFNAR1 deficiency ameliorated HIVgp120-induced microgliosis primarily in the cerebral cortex and to a lesser extent in the hippocampus but had only a minor effect on astrocytosis, diminishing GFAP immunoreactivity in the cerebral cortex but not in the hippocampus. However, the ablation of IFNAR1 reduced overall the number of microglia in both brain regions and immunoreactivity for GFAP in the hippocampus in the absence of HIVgp120. Strikingly, none of the effects on glial cells were sex-dependent, and the apparently reduced microgliosis in the cerebral cortex was not associated with the protection of presynaptic terminals. The lack of a protective effect in the context of diminished microgliosis differed from our observations in CCR5-deficient HIVgp120tg mice [15]. The divergent findings could reflect a more direct influence of CCR5 on neurotoxic microglial activity than of IFNAR1 [16]. On the other hand, the lack of a major effect of IFNAR1 deficiency on astrocytosis in the presence of HIVgp120 is in line with our earlier findings that GFAP immunoreactivity is not indicative of neuronal injury or protection [15, 16, 61].

Since the hippocampus in HIVgp120-expressing mice appeared to be more affected by IFNAR1 than the cerebral cortex, we assessed the expression of the viral envelope itself and several factors that have been implicated in the regulation of HIV-1 neurotoxicity. The knockout of IFNAR1 was associated with a more than a twofold increase of RNA expression of the viral envelope protein but only in males. CCL2, a chemoattractant for microglia and macrophages, was significantly upregulated in HIVgp120tg brains in a sex-independent fashion [15] but IFNAR1 ablation affected it in a sex-dependent manner with opposing results, upregulation in males and downregulation in females. While the upregulation in males seems to fit that of the viral envelope protein and correlated with damage to presynaptic terminals in males versus protection in females, the finding was also surprising in that microglial cell numbers lacked sexual dimorphism. A possible explanation is that CCL2 may not only influence the number of microglia in the hippocampus but also other mechanisms of microglial contribution to HIV-1 neurotoxicity that involve IFNAR1 and remain to be characterized in more detail. Interestingly, CCL5 mRNA was more upregulated in HIVgp120tg brains of males than females compared to WT control, but IFNAR1-deficiency reduced the levels only in males to those in females. While CCL5 can be neuroprotective, the endogenous levels in the HIVgp120tg hippocampus appear to be insufficient to protect presynaptic terminals [32, 33].

The sexual dimorphism observed with CCL2 and CCL5 was completely absent in the mRNA expression for CCL3, and CCL4, CCR5, and CXCR4, as well as the chemokines CXCL10, CXCL11, and CXCL12 and the ISG MX1. The upregulation of MX1 and CXCL11 at mRNA and of STAT1 at the protein level in the HIVgp120tg hippocampus as well as the complete abrogation of the increases in the absence of IFNAR1 confirmed the type I IFN response we previously observed in this model system of HIV-associated brain injury [15, 16]. In addition, IFNAR1 affected mRNA expression for CCL3 and CCL4, CXCL10 and CXCR4 in HIVgp120tg but not in the control hippocampus. We have shown that CCL4 can abrogate neurotoxicity of HIVgp120, and thus, the chemokine’s upregulation may contribute to the protection of neuronal dendrites and presynaptic terminals [16, 32, 33]. Similarly, the downregulation of CXCR4, the co-receptor for the viral envelope protein gp120 expressed as transgene may play a part in the observed neuroprotection. While sex-dependent differences in the course of HIV-1 disease have long been recognized, the reasons for the sexual dimorphism in the injury of presynaptic terminals require further investigation [56].

Along the lines of sexual dimorphism of HIV-associated neuronal damage, the expression of neurotransmission-related genes in the hippocampus was clearly affected by HIVgp120 and IFNAR1 in a sex-dependent fashion. Pre- and post-synaptic components of the glutamate-, GABA-, and dopamine- and serotonergic systems were all affected by the presence of the neurotoxic viral envelope protein. The genetic ablation of IFNAR1 also influenced the expression of pre- and post-synaptic factors except for males in the absence of HIVgp120. IFNAR1KO males differentially regulated only one post-synaptic GABA- and two serotonin receptors (Gabrb1, Htr2c, and Htr4) in association with impaired spatial memory. The specific contributions of these molecules to the behavioral phenotype warrant further investigation. Previous studies by us and others have demonstrated that HIVgp120 toxicity is linked to aberrant glutamate release and excitotoxic neuronal damage but also perturbation of the dopaminergic system [8, 13, 18, 62]. Here, we focused on the effect of IFNAR1 deletion on major neurotransmission systems in the hippocampus in the presence and absence of HIVgp120. The neurotransmission-related gene expression pattern indicated that most significantly affected mRNAs were downregulated in the HIVgp120 hippocampus whether or not IFNAR1 was knocked out. However, the expression patterns in IFNAR1KO × gp120 and HIVgp120tg mice differed, suggesting that protection of neuronal dendrites and partial rescue of presynaptic terminals was not associated with a restoration of normal baseline neurotransmission but rather alternative, apparently compensatory configurations of pre- and postsynaptic components. Bioinformatics utilizing IPA software identified gene networks and biological functions that were affected by HIVgp120 and modified by IFNAR1, such as long-term potentiation, synaptic transmission, and growth of neurites. The major differences between males and females due to IFNAR1 occurred in long-term potentiation and synaptic transmission, which is in line with the more pronounced partial protection conferred by IFNAR1 deficiency to HIVgp120tg females. The similarity regarding the growth of neurites fits with the uniform protection of MAP2^+^ dendrites in both sexes of IFNAR1KO × gp120 mice.

The functional gene networks identified by IPA further displayed the differences between males and females and the genotypes of HIVgp120, IFNAR1KO, and IFNAR1KO × gp120 mice in terms of the regulation of neurotransmission related genes. However, the highest scoring gene networks showing changes driven by HIVgp120 shared downregulation of MAPK1/ERK and CREB1 in females and males, which is in line with the impairment of memory and synaptic injury [63, 64]. Moreover, the networks indicated CREB1 to be restored in the absence of IFNAR1, which fits with the protection of synapses and memory function. But the mechanisms of the sexual dimorphism that underlie the refractory injury in males remain to be elucidated. In any case, IPA scored CREB binding protein (CREBBP) as the major upstream regulator in an overall inhibited CREB1 pathway. Interestingly, in the IFNAR1KO genotype, CREB1 is only downregulated in females, which could explain why the animals show a reduction of SYP^+^ pre-synaptic terminals. However, our behavioral data suggest that this synaptic decline may not be sufficient to compromise recognition or spatial memory. The fact that IFNAR1KO males show normal presynaptic terminals and intact recognition memory while displaying an impairment of spatial memory implies a role for disturbing factors beyond downregulation of CREB1.

Besides the level of expression, the biological activity of CREB1 is regulated by protein kinases, most prominently among them ERK1/2 (MAPK1/3) and p38 MAPK (MAPK14) [44]. However, in contrast to the expression of neurotransmission-related genes, our data indicated that signaling via MAPKs was sex-independent. The reduction of active, phosphorylated ERK1/2 in the hippocampus of the HIVgp120tg brain, which is abrogated in the IFNAR1KO, fits with a role of this kinase and CREB1 in neuronal injury and protection of neuronal MAP2^+^ dendrites. This finding is also in line with previous studies by others showing that activation of ERK1/2 is a crucial signaling pathway for protection against neurotoxicity of HIVgp120 [43]. Similarly, the increase of active, phosphorylated, and total p38 MAPK protein in the cerebral cortex in association with HIVgp120 neurotoxicity, and its amelioration in the IFNAR1KO is in line with the protection of neuronal structure and function. In contrast, active and total p38 MAPK is not elevated above baseline in the hippocampus of HIVgp120tg mice. While an elevation of p38 MAPK activity in the hippocampus could have occurred temporarily as recently shown in an epilepsy model [65], a chronic elevation may not be required for HIVgp120 neurotoxicity. Even if the activity of p38 MAPK remains unchanged, a reduction of ERK activity still alters the balance of the two MAPK pathways which apparently suffices for the neurotoxicity seen in HIVgp120tg hippocampus. However, the finding that SYP^+^ presynaptic terminals are only partially protected and primarily in females, and spatial memory is only normalized in females suggests a crucial role for other, sex-dependent, and IFNAR1-dependent factors in HIV-induced brain injury that remain to be elucidated.

Neurochemical sexual dimorphism has been identified in several neurological diseases and mental disorders that differentially affect men and women [66]. Moreover, women living with HIV show higher immune activation with subsequent faster progression towards AIDS [56]. Animal models can provide important information and help answer questions regarding sex as a biological factor. Here, we report that recognition memory, spatial learning, pre-synaptic terminals, chemokine expression, and expression of genes related to neurotransmission, all revealed sex-dependent differences in HIVgp120tg animals. Although IFNAR1 deficiency resulted in a more pronounced neuroprotective effect in female HIVgp120tg mice compared to males, we observed that p38 MAPK and ERK play critical roles in neuronal injury that seem to be sex-independent. Of note, HIVgp120tg females displayed impaired recognition memory, via mechanisms that are incompletely understood, this finding is in line with previous work in HIV-1 Tg26 transgenic mice, a HIV-1 transgenic rat, and HIVgp120tg mice [58, 67, 68]. The impact of sex on neuroprotection observed here emphasizes the importance of understanding sexual dimorphism in HAND in order to develop new therapeutic approaches.

The pivotal role of IFNAR1 is also reflected in our recent finding that treatment with IFNβ prevented in vitro and in vivo neuronal injury associated with HIVgp120 expression via a mechanism that required IFNAR1 and the β-chemokine CCL4 [16]. Thus, IFNAR1 can contribute to protection if a protective ligand is available at a sufficient concentration and there is no reason to believe that this should not also work in PLWH who are on cART and yet develop HAND. However, there is still surprisingly little known about the role of the IFN system in the brain of PLWH with and without cART. Analysis of cerebrospinal fluid from PLWH and post mortem brain specimen support a role for IFNs in the HIV-infected brain [69, 70]. Our present study indicates that in the absence of protective amounts of IFNβ, IFNAR1 can contribute to neuronal injury in the presence of viral envelope expression. It remains to be elucidated if the baseline expression of IFNα promotes neuronal injury. In any case, even virally suppressed PLWH on cART seem to show signs of chronic inflammation. Since IFNβ is approved by the US Food and Drug Administration (FDA) as a treatment for a neuroinflammatory disease, multiple sclerosis (MS), we believe IFNβ may have therapeutic potential. Another recent study in HIV-1 infected humanized mice found that IFNAR1 blockade rescued both total human T cell and HIV-specific T cell numbers despite elevated viral replication and immune activation [71]. Moreover, Zhen et al. reported that blocking IFNAR2 during the chronic phase of HIV infection led to decreased viral replication, diminished HIV-1-driven immune activation, and restored HIV-1 specific CD8^+^ T cell function [72]. Moreover, the same group showed that cART in combination with IFNAR2 blockade accelerated viral suppression and more efficiently reduced the persistent HIV reservoir compared to cART alone [72]. These and our findings strongly suggest that IFNAR1 and IFNAR2 can provide new therapeutic avenues in the presence and absence of cART.

## Conclusion

In conclusion, our study suggests that IFNAR1 plays a pivotal role in both sex-dependent and independent processes of neuronal injury and behavioral impairment triggered by HIV-1. Behaviorally, we observed that IFNAR1 deficiency rescues spatial learning and recognition memory of HIVgp120tg females. Our data strongly supports that the absence of IFNAR1 in HIVgp120tg mice can rescue the mice from loss of MAP^+^ neuronal dendrite, SYP^+^ presynaptic terminals, microgliosis, and potentially provide neuroprotection against HIVgp120-induced neurotoxicity. Mechanistically, results indicate that knocking down IFNAR1 limits the activation of p38 MAPK in the cortex and restores ERK activity in the hippocampus of the HIVgp120tg brain providing us with an insight that the partial neuroprotection may be driven by these MAPK activities. Thus, future experiments along those lines may provide new therapeutic avenues for HIV-induced brain injuries.

## Supplementary information

**Additional file 1. **Behavior assessment of WT, HIVgp120tg, IFNAR1KO and IFNAR1KO-gp120 mice. Optomotor test for vision (**A**); Barnes maze test: The latencies to enter the escape hole over 4 days of acquisition **(B)**; and the number of errors made to enter the correct escape hole over 4 days of training session (**C**). Statistical analysis was performed as described in the methods section. Genotypes: WT (WT), HIVgp120tg (GP), IFNAR1KO (KO) and IFNAR1KO x gp120 (KOGP). Values are presented in combined box-dot plots with the 25^th^ and 75^th^ percentiles (**A**) or line graphs (**B**, **C**). In box-dot plots, the middle line of the box shows the median, and the mean is indicated by a ‘+’; ANOVA and Tukey’s HSD post hoc test; n = 17-27 animals (males and females) per group/genotype; n.s., not significant)

**Additional file 2.** IFNAR1 deficiency does not alter the expression of JNK in cortex and hippocampus of HIVgp120 mice. The protein expression of phospho-JNK and JNK were assessed using immunoblotting. Representative western blot images and densitometry analysis of phospho-JNK and JNK normalized to GAPDH in the cortex (**A** and **B**) and hippocampus (**C** and **D**). Genotypes: WT (WT), HIVgp120tg (GP), IFNAR1KO (KO) and IFNAR1KO x gp120 (KOGP). Values are presented in combined box-dot plots with the 25^th^ and 75^th^ percentiles. The middle line of the box shows the median, and the mean is indicated by a ‘+’; ANOVA and Tukey’s HSD post hoc test; n.s., not significant; n = 6 animals (3 males and 3 females) per group/genotype

## Data Availability

No databases or software programs or constructs were generated in this study. The new mouse line and data generated during the study are available from the corresponding author upon a reasonable request. The material information and protocol request should be addressed to H.S., R.M., or M.K.

## Abbreviations

ANOVA: Analysis of variance
Ab: Antibodies
Actβ: Actin beta
BBB: Blood-brain barrier
BCA: Bicinchoninic acid
BM: Barnes maze test
BSA: Bovine serum albumin
cART: Combination antiretroviral therapy
CNS: Central nervous system
CREB: cAMP-response element-binding protein
DMSO: Dimethyl sulfoxide
DS: Dopamine/serotonin
ERK: Extracellularly regulated kinase
FDA: Food and Drug Administration
GAPDH: Glyceraldehyde-3-phosphate dehydrogenase
GFAP: Glial fibrillary acidic protein
GG: GABA/glutamate
Gusβ: Glucuronidase beta
HAD: HIV-associated dementia
HAND: HIV-associated neurocognitive disorders
HIVE: HIV-encephalitis
HIVgp120: HIV envelope glycoprotein gp120
Hsp90ab1: Heat shock protein 90 alpha (cytosolic) class B member 1
Iba1: Ionized calcium-binding adapter molecule 1
IFNs: Interferons
IFNAR: Interferon type 1 (IFN-α/β) receptor
IFNAR1KO: Genetic knockout of IFNAR1
IPA: Ingenuity pathway analysis
ISGs: Interferon-stimulated genes
JNK: cJun N-terminal kinase
LDT: Light/dark transfer
LMA: Locomotor activity
mAB: Monoclonal antibody
MAP-2: Microtubule-associated protein 2
MAPK: Mitogen-activated protein kinases
NOR: Novel object recognition
OM: Optomotor
PLWH: People living with HIV
SIV: Simian immunodeficiency virus
SFLI: Sum of fluorescence intensities
STAT1: Signal transducer and activator of transcription 1
SYP: Synaptophysin
tg: Transgenic
WT: Wild-type
