# Supplementary figures and images for: A pivotal role for Interferon-α receptor-1 in neuronal injury induced by HIV-1
Source: J Neuroinflammation. 2020 Jul 29;17:226. doi: 10.1186/s12974-020-01894-2 (PMC7388458; doi:10.1186/s12974-020-01894-2)

A. Optomotor

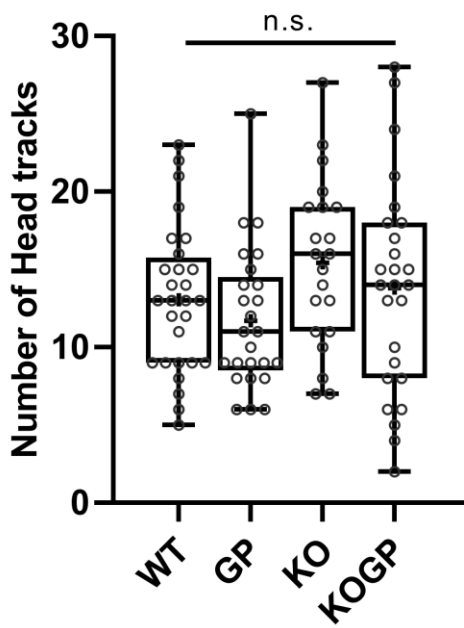

B. Barnes Maze: Errors

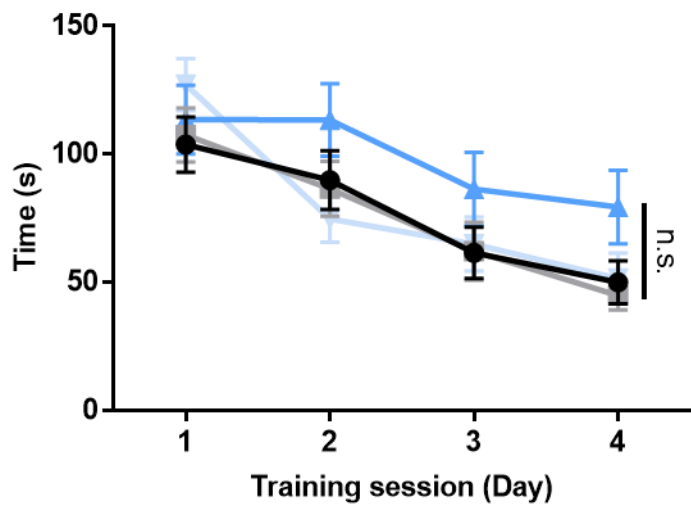

C. Barnes Maze: Latencies to Escape

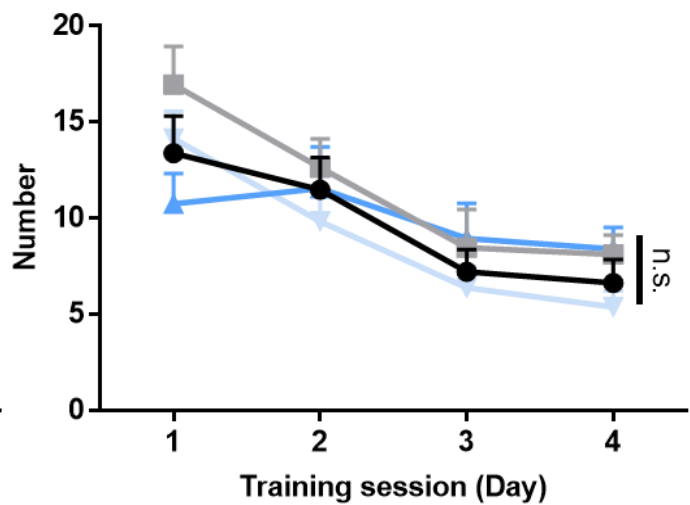

● WT    ■ gp120    ▲ IFNAR1KO    ▼ IFNAR1KO gp120

Supplement: Supplementary file 1 — Additional file 1. Behavior assessment of WT, HIVgp120tg, IFNAR1KO and IFNAR1KO-gp120 mice. Optomotor test for vision (A); Barnes maze test: The latencies to enter the escape hole over 4 days of acquisition (B); and the number of errors made to enter the correct escape hole over 4 days of training session (C). Statistical analysis was performed as described in the methods section. Genotypes: WT (WT), HIVgp120tg (GP), IFNAR1KO (KO) and IFNAR1KO x gp120 (KOGP). Values are presented in combined box-dot plots with the 25th and 75th percentiles (A) or line graphs (B, C). In box-dot plots, the middle line of the box shows the median, and the mean is indicated by a ‘+’; ANOVA and Tukey’s HSD post hoc test; n = 17-27 animals (males and females) per group/genotype; n.s., not significant) [file 12974_2020_1894_MOESM1_ESM.pdf]

## A. Cortex

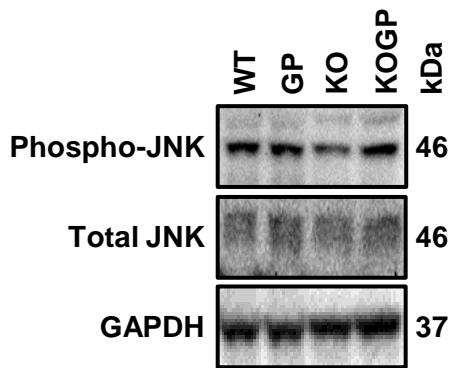

## B.

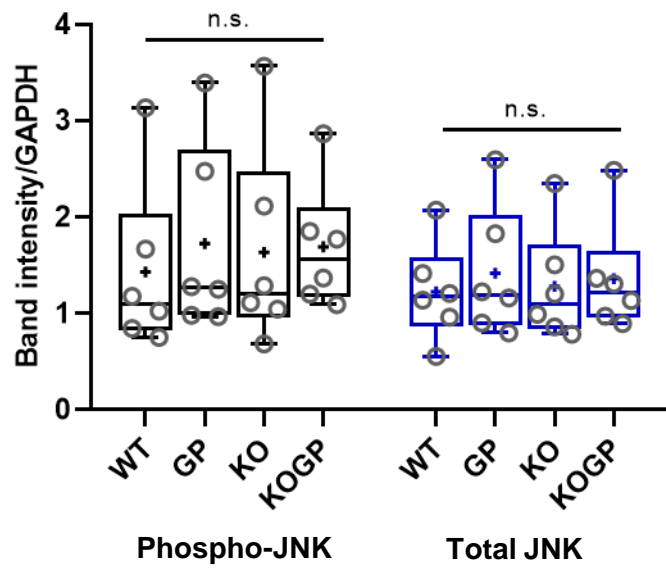

## C. Hippocampus

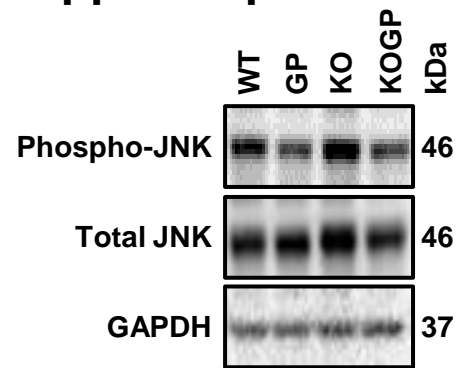

## D.

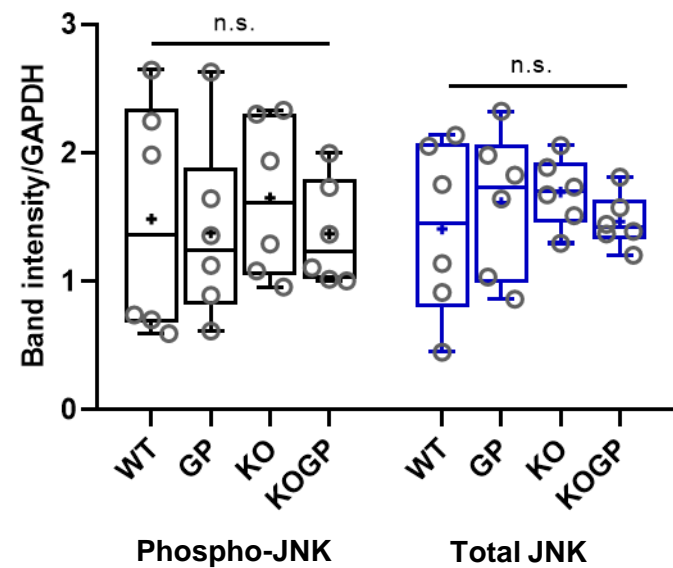

Supplement: Supplementary file 2 — Additional file 2. IFNAR1 deficiency does not alter the expression of JNK in cortex and hippocampus of HIVgp120 mice. The protein expression of phospho-JNK and JNK were assessed using immunoblotting. Representative western blot images and densitometry analysis of phospho-JNK and JNK normalized to GAPDH in the cortex (A and B) and hippocampus (C and D). Genotypes: WT (WT), HIVgp120tg (GP), IFNAR1KO (KO) and IFNAR1KO x gp120 (KOGP). Values are presented in combined box-dot plots with the 25th and 75th percentiles. The middle line of the box shows the median, and the mean is indicated by a ‘+’; ANOVA and Tukey’s HSD post hoc test; n.s., not significant; n = 6 animals (3 males and 3 females) per group/genotype [file 12974_2020_1894_MOESM2_ESM.pdf]
